# Reporter gene-engineering of human induced pluripotent stem cells during differentiation renders *in vivo* traceable hepatocyte-like cells accessible

**DOI:** 10.1016/j.scr.2019.101599

**Published:** 2019-12

**Authors:** Candice Ashmore-Harris, Samuel JI Blackford, Benjamin Grimsdell, Ewelina Kurtys, Marlies C Glatz, Tamir S Rashid, Gilbert O Fruhwirth

**Affiliations:** a Imaging Therapy and Cancer Group, Department of Imaging Chemistry and Biology, School of Biomedical Engineering and Imaging Sciences, St Thomas’ Hospital, King's College London (KCL), London, SE1 7EH, UK; b Centre for Stem Cells and Regenerative Medicine, School of Basic and Medical Biosciences, Guy's Hospital, KCL, London SE1 9RT, UK; c Centre for Human and Applied Physiological Sciences, School of Basic and Medical Biosciences, Shepherd's House, King's College London, SE1 1UL, UK; d Institute of Liver Studies, King's College Hospital NHS Foundation Trust, London SE5 9RS, UK

**Keywords:** Cell tracking, Hepatocyte-like cells, Human sodium iodide symporter, Induced pluripotent stem cells, Lentivirus, Radionuclide imaging

## Abstract

Primary hepatocyte transplantation (HTx) is a safe cell therapy for patients with liver disease, but wider application is circumvented by poor cell engraftment due to limitations in hepatocyte quality and transplantation strategies. Hepatocyte-like cells (HLCs) derived from human induced pluripotent stem cells (hiPSC) are considered a promising alternative but also require optimisation of transplantation and are often transplanted prior to full maturation. Whole-body *in vivo* imaging would be highly beneficial to assess engraftment non-invasively and monitor the transplanted cells in the short and long-term.

Here we report a lentiviral transduction approach designed to engineer hiPSC-derived HLCs during differentiation. This strategy resulted in the successful production of sodium iodide symporter (NIS)-expressing HLCs that were functionally characterised, transplanted into mice, and subsequently imaged using radionuclide tomography.

## Highlights

• iPSC-derived hepatocyte-like cells (HLCs) rendered traceable *in vivo.*
• Reproducible lentivirus-based gene transfer during the differentiation process.
• Protocol and reporter expression did not negatively impact on HLC maturation.
• Proof-of-principle shown for whole-body SPECT/CT-afforded HLC *in vivo* tracking.

## Graphical abstract

**Figure:**
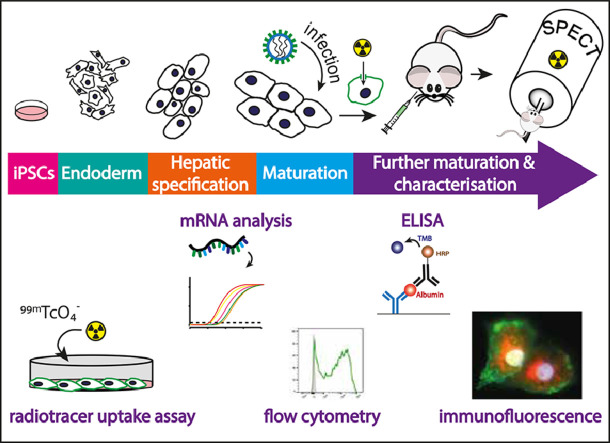

## 1 Introduction

Orthotopic liver transplantation (OLT) is the core treatment for end-stage liver disease and some metabolic disorders but patient need consistently exceeds donor organ availability. Primary hepatocyte transplantation (HTx) is a well-established, safe cell therapy benefitting from reduced invasiveness, retention of the native liver architecture and offering repeat use of cryopreserved donor cells (Forbes et al., 2015). Whilst HTx tends to result in relatively short-term improvement in hepatic function or graft rejection, preconditioning regimes have shown improved engraftment pre-clinically, but require optimisation (Soltys et al., 2017). Success of HTx engraftment has also been strongly linked to cell quality (Donato et al., 2008; Ibars et al., 2016), yet it relies on hepatocytes isolated from livers rejected for OLT (*e.g.* geriatric donors, prolonged ischaemia, fatty livers). To circumvent HTx quality and supply limitations, human induced pluripotent stem cell (hiPSC)-derived hepatocytes have been considered a potential substitute. Several protocols were developed to differentiate hiPSCs into hepatocyte-like cells (HLCs) (Hannan et al., 2013; Rashid et al., 2010; Si-Tayeb et al., 2010; Song et al., 2009). Moreover, clinical-grade hiPSC lines capable of differentiation into HLCs (Baghbaderani et al., 2015; Blackford et al., 2018; Wang et al., 2015) accompanied by protocols demonstrating clinically relevant scalable HLC or liver organoid production (Takebe et al., 2017; Wang et al., 2017; Yamashita et al., 2018) significantly progressed the clinical realisation of HLC therapies.

There is currently no standard protocol for preclinical HLC transplantation *in vivo*, with variations in transplantation site, cell format (single cell suspensions *vs.* novel formats) and stage of differentiation at transplant reported (Asgari et al., 2013; Chen et al., 2015; Nagamoto et al., 2016; Pettinato et al., 2016; Takayama et al., 2017; Takebe et al., 2017). Post-transplant evaluations typically rely on blood/serum analyses for soluble factors and liver enzyme activity, offering no information on the *in vivo* location of transplanted cells and providing only indirect viability information. Alternatively, histology of biopsied tissues demonstrates localised engraftment but is invasive and a risk to both the host and transplanted cells. The option to track engrafted cells would be highly beneficial. Non-invasive whole-body imaging would provide spatiotemporal information about their *in vivo* location and viability both short and long-term and allow quantitative comparison between different transplantation strategies.

*In vivo* cell tracking can be achieved by directly labelling cells or by employing reporter gene technology with the latter offering several advantages; (i) the observation period is independent of the contrast agent, *i.e.* not limited by label efflux or the half-life of a radioisotope; (ii) genetic encoding avoids label dilution phenomena and better reflects cell viability, and also (iii) circumvents complex direct cell labelling procedures and associated toxicities (Fruhwirth et al., 2018; Volpe et al., 2018). Its drawback is the need for genetic engineering. Host reporter proteins are preferable to foreign reporters, which are prone to recognition/destruction by an intact immune system. Importantly, host reporters should not be expressed in the transplanted tissue of interest, and only in a limited number of other host tissues, ideally at low levels to ensure favourable contrast during imaging.

The human sodium iodide symporter (hNIS) is a transmembrane glycoprotein that has been exploited as a radionuclide reporter gene for both single photon computed tomography (SPECT) and positron emission tomography (PET) in a variety of cell tracking settings; including cancer metastasis (Diocou et al., 2017; Fruhwirth et al., 2014; Volpe et al., 2018), migration of mesenchymal stem cells (Dwyer et al., 2011), tracking of hiPSC and cardiac stem cell myocardial infarction models (Templin et al., 2012; Terrovitis et al., 2008), and embryonic stem cell-caused teratomas (Wolfs et al., 2017). hNIS is endogenously expressed at high levels in the thyroid gland and at lower levels in few extrathyroidal tissues (salivary glands, mammary glands, stomach and small intestine) (Portulano et al., 2014). Its function depends on an intact Na^+^/K^+^ gradient, driven by cellular ATP, and thus it sensitively reports only live cells.

Here, our aim was to develop a protocol for the generation of *in vivo* traceable HLCs during differentiation, to enable compatibility with the range of transplantation protocols currently utilised in the field and provide a non-invasive approach to optimise HLC engraftment protocols in the future. We assessed the impact of lentiviral gene transfer on HLC maturation and provided proof-of-principle *in vivo* detection of resultant traceable hNIS-mGFP^+^ HLCs by SPECT/CT imaging.

## 2 Material and methods

### 2.1 Reagents and chemicals

Purchased from Sigma, Thermo-Fisher, Gibco or StemCell Technologies, unless otherwise stated. All cell lines including hiPSC lines have been previously described and were grown as recommended (*cf.* Supplement). Standard *in vitro* methodologies including lentivirus (LV) production, flow cytometry, gene expression analysis, secreted albumin and cell viability determinations, cellular radiotracer uptake, and immunofluorescence staining are detailed in the Supplement.

### 2.2 Cell differentiation

hiPSC colonies from the patient-derived A1ATD^z/z^ (Rashid et al., 2010; Yusa et al., 2011) or the cGMP derived CGT-RCiB-10 (WCB) hiPSC line (Cell and Gene Therapy Catapult, UK), herein referred to as A1AT and CGT10 respectively, were differentiated under hypoxic conditions (5% (v/v) O_2_, 5% (v/v) CO_2_, 37°C) via serial introduction of small molecules and growth factors as previously described (Blackford et al., 2018).

### 2.3 Cell transduction

Cells were washed with PBS. Viral particles with an estimated MOI of 5 (based on 2 × 10^6^ cells expected per 10 cm dish) were diluted in hepatocyte maturation media and added dropwise to cover cells (3 mL media/10 cm dish). Dishes were left at room temperature for 15 min. 1.5mLfresh medium was added to each dish and cells were incubated overnight in hypoxic conditions (5% CO_2_, 5% O_2_). 24 h later 1.5 ml fresh medium was added and after 48 h the cells were washed and lifted for either re-seeding/*in vitro* maturation, *in vivo* transplantation, or flow cytometric analysis.

### 2.4 Cell preparation for *in vivo* experiments

For intraperitoneal injection of pre-labelled cells, cells were first radiolabelled as for *in vitro* uptake assays with 100 kBq ^99m^TcO_4_^−^/mL. Cells were subsequently washed twice with PBS^++^, lifted using TrypLE and resuspended in PBS^++^ at 10^7^cells/100 µL. Radiolabelled cells were used immediately for injection into animals.

### 2.5 Animals

NOD.Cg-*Prkdc^scid^Il2rg*^*tm1Wjl*^/SzJ (NSG; Charles River UK) were maintained under sterile conditions with food and water available *ad libitum*. All procedures were performed in accordance with all legal, ethical, and institutional requirements.

### 2.6 *In vivo* imaging and image analysis

Mice were anesthetized with 2% (v/v) isoflurane in pure oxygen and injected either with *in vitro* labelled HLCs intrahepatically (to set up the animal model) and/or with 30 MBq of the NIS-radiotracer ^99m^TcO_4_^−^ (in 100 µL sterile PBS) i/v (to enable *in vivo* NIS imaging). Animals remained under anesthesia from radiotracer administration throughout SPECT/CT scanning. Immediately after intrahepatic HLC injection or 45 min after i/v radiotracer administration SPECT imaging was performed over a period of 60 min using a preclinical Nanoscan SPECT/CT Silver Upgrade instrument (Mediso) equipped with 1 mm collimators. CT image acquisition (55 kVp tube voltage, 1200 ms exposure time in 360 projections) was performed after tracer administration but before SPECT scanning to reduce overall anesthesia duration. Repeat imaging with the same radiotracer was not affected by tracer amounts from the first imaging session under these conditions as previously demonstrated (Diocou et al., 2017).

All SPECT/CT data sets were reconstructed using a Monte Carlo-based full 3D iterative algorithm (Tera-Tomo; Mediso). Corrections for attenuation, detector dead time, and radioisotope decay were in place as needed. All images were analysed using VivoQuant software (inviCRO), enabling the co-registration of SPECT and CT images and delineation of regions of interest (ROIs) for quantification of radioactivity. As background can vary in different locations *in vivo* it is important to consider local/regional thresholding and segmentation. We employed the 3D implementation of Otsu's method (Otsu, 1979) for rendering volumes with radioactivity counts above background. The total activity in the whole animal (excluding the tail) at the time of tracer administration was defined as the injected dose (ID). Radioactivity in each ROI was quantified using VivoQuant software and expressed as standard uptake value (SUV).

### 2.7 Liver histology

Harvested livers were separated into individual lobes, embedded in optimal cutting temperature (OCT) medium and frozen in isopentane pre-cooled over liquid nitrogen and stored at −80°C. 10 µm sections were cut with a Cryomatic cryostat (Bright Ltd, Huntingdon, UK), placed on polysine-coated slides and fixed in 4% paraformaldehyde (15 min at room temperature (RT)), washed thrice in PBS before blocked for 1 h (PBS containing 1.5% bovine serum albumin, 3% donkey serum, 0.1% Triton-X). Sections were incubated with goat anti-human albumin antibody (Bethyl laboratories #A80-129A, 10 µg/mL) overnight at 4°C, washed thrice in PBS-T (PBS containing 0.1% Tween-20), and stained with donkey anti-goat secondary antibody conjugated to Cy3 (Jackson/Stratech, 705–165–147, 2 µg/mL) for 45 min at RT. After three PBS-T washes, nuclei were stained with Hoechst 33,342 (1 µg/mL) for 10 min at RT, washed another three times in PBS-T, rinsed twice in deionized water and mounted (10%%(w/v) Mowiol 4–88 containing 2.5%(w/v) DABCO). Images were taken on a NIKON Eclipse Ti-E inverted fluorescence microscope equipped with a 20x NA 0.45 objective lens and filter sets appropriate for imaging Hoechst 33342 (cell nuclei) and Cy3 using NIS Elements acquisition software; for image processing ImageJ software v1.41 (NIH) was used.

## 3 Results

### 3.1 Generation of *in vivo* traceable HLCs by transduction during differentiation

**Fig. 1:**
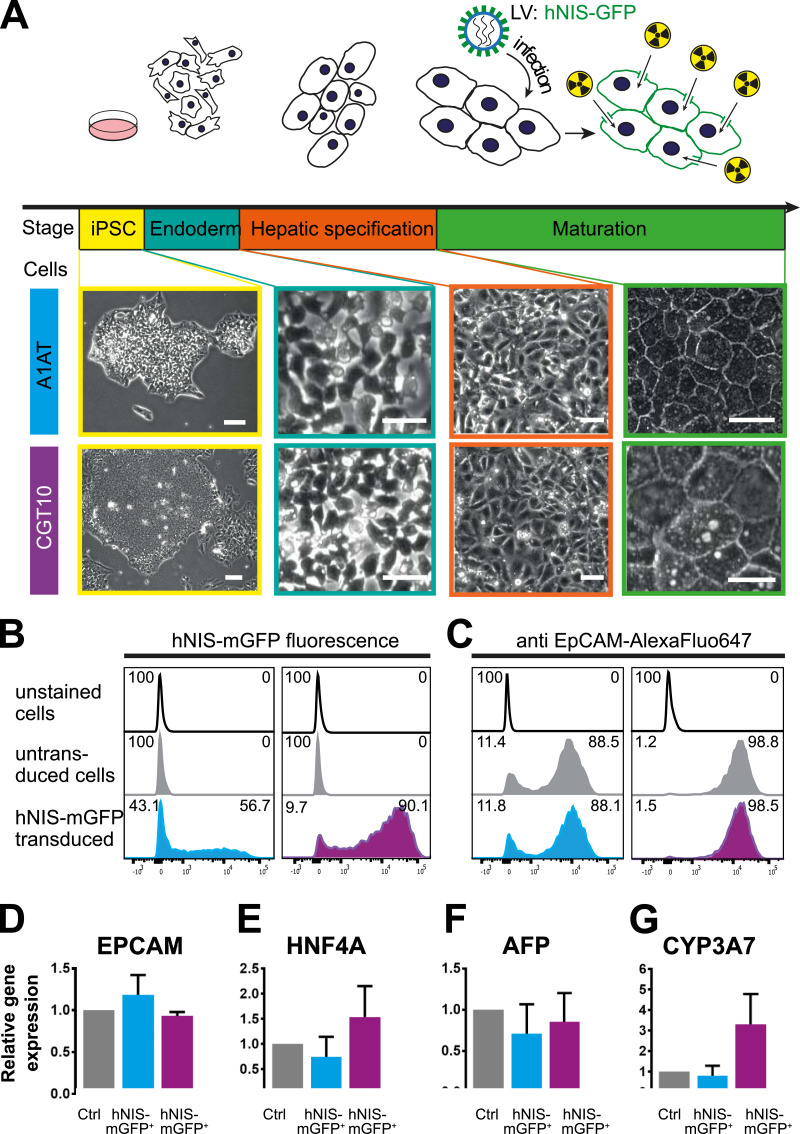
Comparison of untransduced and hNIS-mGFP^+^ HLCs 2 days after transduction | (A) Scheme depicting the differentiation stages of iPSCs to HLCs (top) combined with micrographs showing the morphologies of both A1AT and CGT10 HLCs at the corresponding differentiation stages. Representative live cell flow cytometric analyses of differentiating HLCs on day 20 showing (B) GFP signals in transduced HLCs and (C) comparable surface marker expression of the hepatic progenitor marker EpCAM. mRNA expression analysis (by qRT-PCR) of an immature hepatocyte gene panel 2 days after transducing the differentiating cells: (D) EpCAM, (E) Hepatic nuclear factor 4α (HNF4A), (F) ɑ-fetoprotein (AFP), and (G) Cytochrome P450 Family 3A7 (CYP3A7). Control and transduced cells were always from the same differentiation batches and values normalised to corresponding control cells. *N* = 3 biological replicates (*i.e.* independent differentiations); error bars are SEM; two-tailed Student's *t*-test was applied (*p* > 0.05 for all).

Established protocols to differentiate hiPSCs into HLCs employ a stepwise approach: induction of endoderm, hepatic specification, hepatoblast expansion and hepatic maturation (Hannan et al., 2013; Rashid et al., 2010; Si-Tayeb et al., 2010; Song et al., 2009). We used two hiPSC lines, A1AT^z/z^ RMA B08 (A1AT) and the cGMP-compliant line CGT-RCiB-10 (CGT10), which differentiated into HLCs adopting the expected morphology throughout (Fig. 1A). Briefly, tightly packed hiPSC colonies with a high nuclear-to-cytoplasm ratio and prominent nucleoli expanded rapidly from initially seeded colonies following induction of endoderm differentiation. Definitive endoderm was stimulated towards hepatic endoderm and the resulting hepatoblast population rapidly expanded and proliferated creating a confluent monolayer. Subsequently, a polyhedral monolayer of immature HLCs with high cytoplasm-to-nuclear ratio was obtained. On day 18, we transduced the differentiating cell lines with LVs containing DNA encoding for the dual-mode radionuclide-fluorescence reporter gene hNIS-mGFP. Transduction at earlier differentiation time points (*e.g.* day 10) was inefficient due to either very low transduction efficiencies (hiPSC stage) or cell loss during transduction in the hepatoblast expansion stage (high cell turnover; Fig. S1).

We validated that differentiation occurred as expected at two differentiation time points and compared transduced cells to non-transduced populations from the same differentiation batch. The first time point was two days post-transduction (day 20) at the immature HLC stage when HLCs are typically passaged onto collagen-I for further maturation *in vitro* or lifted for *in vivo* transplantation. Flow cytometry confirmed hNIS-mGFP expression (Fig. 1B) and comparable expression of the hepatic progenitor marker epithelial cell adhesion molecule relative to untransduced control HLCs (EpCAM; Fig. 1C). Additional markers of foetal hepatocyte development were quantified by quantitative real-time PCR to assess whether the hepatic lineage was retained: ɑ-fetoprotein (AFP), cytochrome P450 Family protein 3A7 (CYP3A7), and hepatic nuclear factor 4α (HNF4A) (Fig. 1D–F). No significant differences between hNIS-mGFP^+^ HLCs and untransduced control HLCs were observed. Similar results were obtained when primary foetal hepatocytes were transduced (Fig. S2). Long-term culture of hNIS-mGFP^+^ A1AT HLCs demonstrated morphology, cell viability, and albumin production to be comparable to untransduced HLCs, as well as reporter expression to be stable for at least 100 days (Fig. S3).

**Fig. 2:**
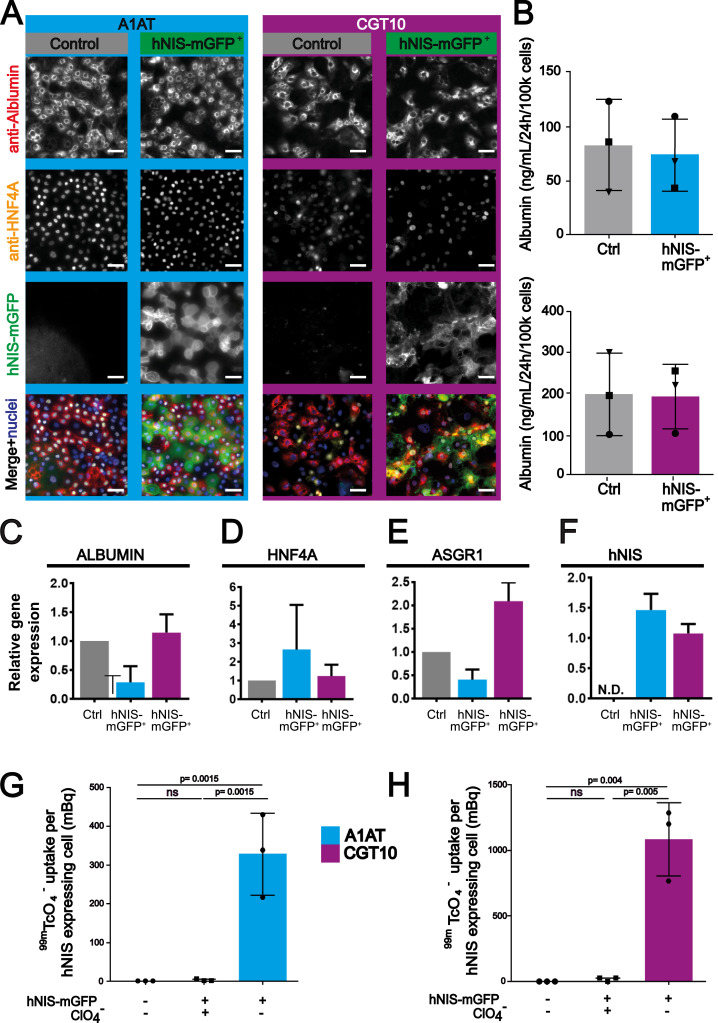
Comparison of untransduced and hNIS-mGFP^+^ HLCs two weeks after transduction | (A) Immunofluorescence micrographs demonstrating hNIS-mGFP^+^cells mature following passage and co-express the gold standard markers for hepatic cells, albumin and HNF4A. Images representative of 3 independent differentiations. Scale bars 100 µm. (B) Secreted albumin concentrations are not significantly different between control and transduced batches. Similarly, no significant differences were detected for (C-E) marker gene expression by qRT-PCR (normalised to corresponding control batches). (F) Day 34 hNIS expression by qRT-PCR relative to expression at day 20 demonstrating expression is stable (G-H) hNIS-mGFP function quantified by ^99m^TcO_4_^−^ uptake relative to untransduced control HLCs. The hNIS co-substrate perchlorate served as a ^99m^TcO_4_^−^ uptake specificity control. *N* = 3 biological replicates (with three (B, G-H) or four (C-F) technical repeats per biological sample); error bars are SEM (B-F) or SD (G-H). Statistical tests were Students’ *t*-test (B-F) or one-way ANOVA with Tukey's multiple comparison correction (G-H); “ns” represents *p*> 0.05.

Following passage of HLCs onto collagen-I, we cultured both hNIS-mGFP^+^ HLCs and untransduced control HLCs for two weeks before assessing whether the transduced cell population retained the capacity to mature. Most cells exhibited the typical polyhedral morphology and cells co-expressing albumin and HNF4A, considered the gold standards for *bona fide* hepatocytes, were seen in abundance along with more immature cells (Fig. 2A). We further observed that the expected membrane expression of hNIS-mGFP was maintained throughout the maturation process with cells capable of co-expressing HNF4A, albumin and hNIS, indicating similar levels of maturation of both populations. To verify this independently, we analysed media concentrations of secreted albumin by ELISA and confirmed them to be similar (Fig. 2B). Moreover, expression levels of *ALBUMIN* and *HNF4ɑ* mRNA were comparable between hNIS-mGFP^+^ and untransduced HLCs (Fig. 2C-D). Additionally, we analysed gene expression of the asialoglycoprotein receptor isoform 1 (ASGR1; Fig. 2E), which is typically found on the cell surface of functional mature HLCs (Peters et al., 2016). All markers we investigated demonstrated that hNIS-mGFP^+^ HLCs and untransduced HLCs did not differ significantly. This provided evidence that our LV transduction strategy did not negatively impact on maturation to the hepatic phenotype in these hiPSC lines. Furthermore, hNIS mRNA expression levels were retained at comparable levels at day 34 relative to day 20 indicating that reporter expression was stable throughout maturation (Fig. 2F).

We next assessed hNIS-mGFP functionality when expressed in transduced HLCs by quantifying cellular uptake of the hNIS radiotracer ^99m^TcO_4_^−^. Both mature (differentiation day 34, *i.e.* two weeks post-transduction) hNIS-mGFP^+^ HLC cell lines readily took up the radiotracer as compared to untransduced cells (Fig. 2G-H). Moreover, ^99m^TcO_4_^−^ uptake was sensitive to competition with the hNIS co-substrate perchlorate, thereby confirming hNIS specificity.

### 3.2 *In vivo* imaging of hNIS-mGFP^+^ HLCs

**Fig. 3:**
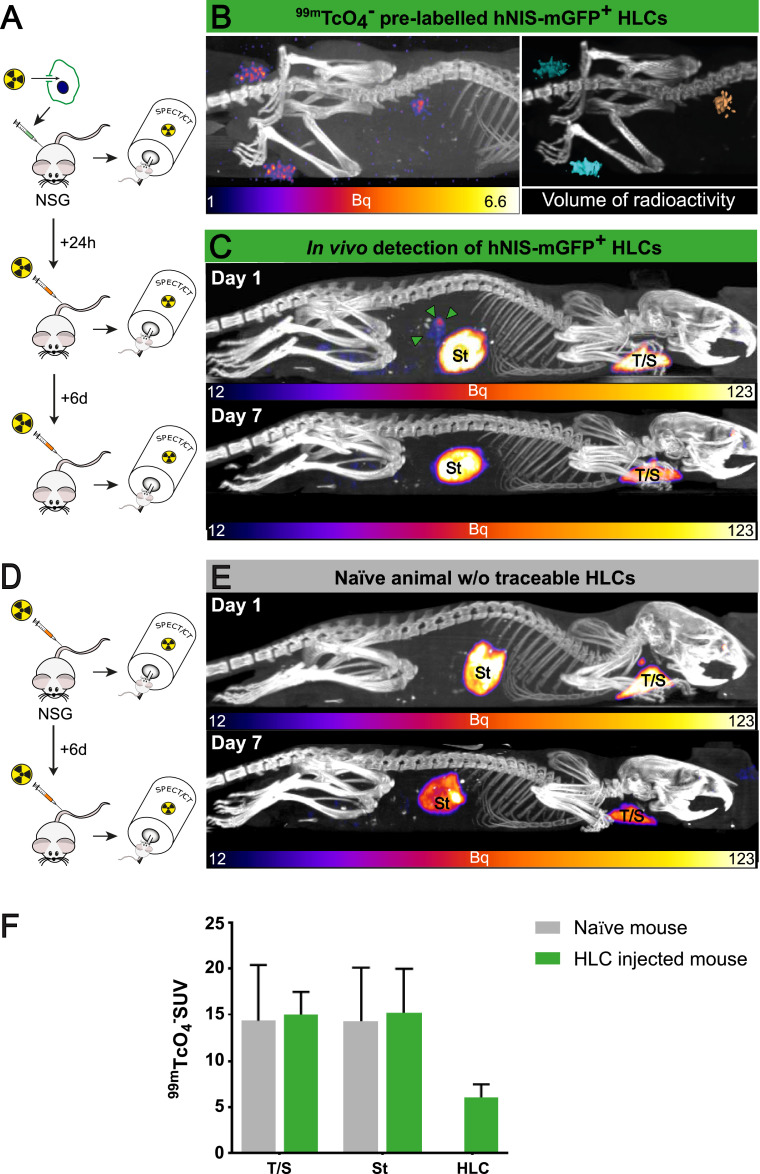
Non-invasive *in vivo* imaging of hNIS-mGFP^+^ CGT10 HLCs | (A) Scheme depicting the experimental set-up; briefly, ^99m^TcO_4_^−^-labelled hNIS-mGFP^+^ HLCs were administered to NSG mice, animals imaged, and after radiotracer decay re-imaged post systemic ^99m^TcO_4_^−^ injection on the next day. (B/left) SPECT/CT maximum intensity projection (MIP) of pre-labelled and intrahepatically administered CGT10 HLC (*cf.* A/top). (B/right) 3D volume rendering of SPECT signals subsequent to local signal/noise thresholding (Otsu method) and overlaid onto CT image (blue=cell pellet phantoms; gold=*in vivo* administered cells). (C) SPECT/CT MIP obtained post systemic ^99m^TcO_4_^−^ injection demonstrating intrahepatic detection of hNIS-mGFP^+^ HLCs 24 h post cell transplantation but cell loss at 7d Endogenous NIS signals correspond to thyroid/salivary glands (T/S) and the stomach (St). (D-E) Naïve animals were SPECT/CT imaged as in C with only endogenous murine NIS signals being evident (St=stomach; T/*S*=thyroid/salivary glands). (F) Image-derived *in vivo* biodistribution data (24 h post administration) corresponding to *n* = 2 naïve control mice and *n* = 4 HLC mice. (For interpretation of the references to colour in this figure legend, the reader is referred to the web version of this article.)

First, we pre-labelled hNIS-mGFP^+^ CGT10 HLCs *in vitro* with ^99m^TcO_4_^−^ (16.2 ± 4.1kBq/5 × 10^6^ cells). The cells were harvested, suspended in PBS to a final volume of 50 µL and injected intra-hepatically into NSG mice, which were imaged by SPECT/CT immediately post-injection (Fig. 3A). The signal of the pre-labelled hNIS-mGFP^+^ CGT10 HLCs was clearly visible at the site of injection. Image quantification revealed a total amount of 5.2 kBq ^99m^Tc in the thresholded and segmented location, which had a total rendered volume of 14.3 mm^3^ (Fig. 3B-C). Notably, we twice used the same amount of hNIS-mGFP^+^ CGT10 HLCs from the same pre-labelled batch, suspended them in a final volume of 50 µL saline in 1.5 mL tubes, but placed them as imaging phantoms alongside the animals’ rear legs (Fig. 3B-C) as quantification controls. The quantified average ^99m^Tc activity in the phantoms was 6.5 kBq within average rendered volumes of 14.8 mm^3^. The ∼18% lower radioactive signal in the segmented image in the mouse as compared to the phantoms indicates cell loss upon *in vivo* injection, most likely due to cell dispersal.

24 h later, the animals were re-imaged by SPECT/CT following intravenous (i/v) ^99m^TcO_4_^−^ administration to verify if intra-hepatically administered hNIS-mGFP^+^ HLCs were detectable *in vivo* without pre-labelling (Fig. 3A). Due to the short half live of ^99m^Tc (τ ==6.01 h), only ∼6% of the radioactivity (0.3 kBq) from *in vitro* labelling was left at the time of i/v administration of ^99m^TcO_4_^−^. Control animals received only the i/v administered radiotracer (Fig. 3D). ^99m^TcO_4_^−^ uptake by hNIS clearly demonstrated reporter function *in vivo* in HLCs and, more importantly, that the administered hNIS-mGFP^+^ CTG10 HLCs were detectable in the liver without interference from any organs endogenously expressing mouse NIS (homologous to hNIS; Fig. 3; (Portulano et al., 2014)). *In vivo* image quantification revealed HLCs uptake of 25.4 kBq ^99m^Tc in the background-corrected and segmented location, which had a total rendered volume of 4.1 mm^3^, a noticeable decrease in size relative to the previous scan. Imaging data demonstrated that hNIS-mGFP^+^ CTG10 HLCs remained alive for at least 24 h post administration, but were undetectable after seven days, most likely due to death in the liver environment.

**Fig. 4:**
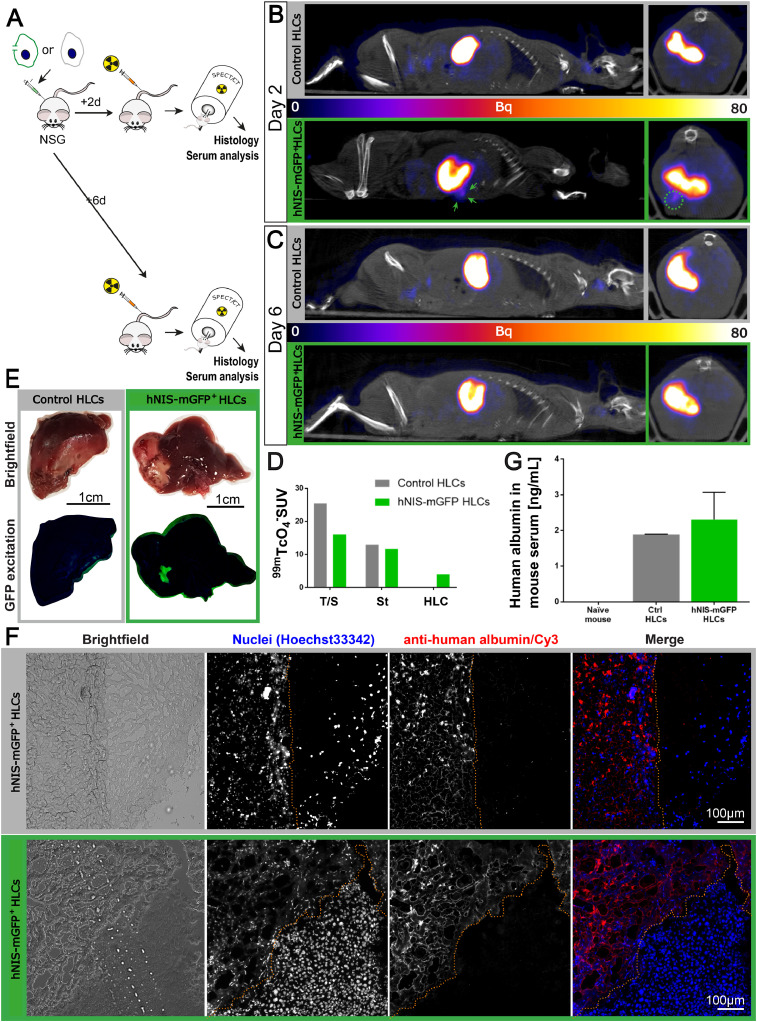
Intra-hepatically transplanted hNIS-mGFP^+^ HLCs behave similarly compared to untransduced HLCs | hNIS-mGFP^+^ and control, non-reporter expressing HLCs from the same batch of differentiation were transplanted intrahepatically at day 23 of differentiation. (A) Scheme of the *in vivo* experiment indicating control or hNIS-mGFP^+^ HLC intrahepatic transplant and ^99m^TcO_4_^−^-afforded NIS imaging sessions by SPECT/CT two days or six days post-transplant. (B/C) SPECT/CT sagittal (left) and transverse (right) slices of transplanted mice imaged two days after transplantation. Expected endogenous signals (from stomach) are visible in both groups of animals. Importantly, hNIS-mGFP^+^ HLCs were detected at the expected location in the liver in corresponding animals two days after transplantation (B/green arrows and circle). However, six days post transplantation, no hNIS-mGFP^+^ HLC were detectable (C). In control animals no signals were recorded at any time, as expected (B/C). (D) Standard uptake values (SUV) from relevant tissues determined from *in vivo* images using VivoQuant (Invicro) software. (E) Representative post-mortem liver lobes from animals two days after transplantation (matching (B)). Areas containing transplanted cells (pale red, brightfield) are visible. Fluorescence excitation (450/20 nm BP) paired with a camera behind an emission filter (500 nm LP) allowed specific visualization of hNIS-mGFP^+^ HLCs, while untransduced control HLCs were not detectable, confirming cell survival and functionality of the hNIS-mGFP^+^ reporter at two days after transplantation. (F) 10 µm liver sections of snap-frozen livers from corresponding livers in B and D were stained for human albumin, indicating presence of human cells alongside mouse liver cells (no human albumin staining, different morphology in brightfield). (G) Mouse serum from animals sacrificed two days after HLC transplantation was subjected to ELISA analysis for human albumin. Even at this early time point after transplantation, ELISA allowed the detection of human serum in both reporter-expressing and untransduced animals, confirming the function of the transplanted cell at this time point. (For interpretation of the references to colour in this figure legend, the reader is referred to the web version of this article.)

To verify that this limited cell survival was not due to reporter expression, hNIS-mGFP^+^ and non-reporter expressing control HLCs from the same batch of differentiation were transplanted intra-hepatically on day 23 of the differentiation protocol. We observed cell survival by SPECT/CT imaging for 2 days post transplantation into the liver in the hNIS-mGFP^+^HLC population (Fig. 4A–C). Following animal sacrifice, we detected in excised livers some hNIS-GFP^+^ HLCs (Fig. 4D) and confirmed this by histology (Fig. 4E; staining for human albumin in sections from the transplant sites). Notably, both control and traceable HLCs were detected by histology at day 2. Importantly, human albumin (albeit at low levels due to the early time point after transplantation) was found in the serum of both animals transplanted with hNIS-mGFP^+^ and control HLCs (Fig. 4F). Radioactivity measurements of the corresponding organs revealed similar levels in organs endogenously expressing NIS and specific uptake in the livers of mice transplanted with hNIS-GFP^+^ HLCs. Similar to the results in Fig. 3, neither hNIS-mGFP^+^ nor control HLCs from the same transplant cohorts were detectable at day 6 (neither by *in vivo* imaging (Fig. 4C) nor by any tissue analyses (data not shown)) indicating that both control and hNIS-mGFP^+^ cells exhibit the same limited survival behaviour *in vivo* in this animal model. Comparatively, similarly engineered liver cancer cells transplanted subcutaneously or intrahepatically and allowed to establish tumours were detectable for at least five weeks (Fig. S4-S5; endpoints determined by animal regulations and not by detectability), validating our *in vivo* tracking approach. This suggests that death of our HLC populations is not due to the method of transplantation, that there is no disadvantage for traceable HLCs, but rather that the long-term tracking capacity of the traceable liver cancer cells is caused by their inherent survival advantage.

## 4 Discussion

Multiple studies, both clinically and preclinically, have aimed to improve the efficacy of HTx therapy (Boudechiche et al., 2015; Nagamoto et al., 2016, 2014; Soltys et al., 2017; Yamanouchi et al., 2009). Typically, analyses of HTx engraftment are restricted to serial biopsies and/or serum samples without the involvement of non-invasive monitoring. This is especially problematic for research on: the impact of the transplant site on graft retention; the optimal timepoint within differentiation for maximal engraftment/proliferation post-transplant; the therapeutic benefit/success of suspension cell transplants over novel formats (*e.g.* hepatocyte sheets, liver organoids); or the benefits of repeat infusions/treatment intervals on graft retention/therapeutic effect.

Here, we demonstrated LV-mediated gene transfer during differentiation, thereby introducing the radionuclide-fluorescence reporter hNIS-mGFP into differentiating immature iPSC-derived HLCs rendering them traceable *in vivo*. As LVs efficiently infect non-dividing cells, we chose to transduce differentiating cells after the key stages of specification and proliferation when HLCs already exhibited the distinctive polyhedral hepatic morphology. Transduction during differentiation renders gene transfer compatible with the range of *in vivo* transplantation protocols in the field, crucially including transplantation of HLCs prior to final maturation allowing completion of maturation post transplantation (*cf.* Introduction). Resultant hNIS-mGFP^+^ HLCs showed no significant phenotypic changes compared to untransduced normally differentiating HLCs (Fig. 1) and continued to mature as expected (Fig. 2).

Lentiviral transduction results in gene transfer that is not site-specific but also not random. It results in polyclonal populations in respect to the genomic insertion position, which represents a safety concern for genetic engineering of stem cell therapies. Notably, lentiviruses were found to be safer than for example conventional γ-retroviruses (Biffi et al., 2011). Currently, lentiviruses are the tool of choice to produce several clinically approved genetically engineered cell therapies (*e.g.* anti-cancer chimeric antigen receptor T cell therapies). Non-viral alternatives have been developed but often suffer from low transfection rates, cell toxicities and/or expression stability (Ramamoorth et al., 2015). Recent genome-editing methodologies (Maeder and Gersbach, 2016) enable site-specific insertion of a reporter and thus have the potential to overcome the safety concerns of viral transduction methodologies. Another aspect is the differentiation status of the therapeutic cells; the more differentiated and hence less pluripotent, the smaller the overall risk associated with viral gene transfer (*cf.* we transduced on day 18 when the differentiating cells were already committed to the hepatic lineage and beyond the hepatoblast expansion stage; Fig. 1). It is noteworthy that we did not observe any teratoma formation, in contrast we observed HLC loss within less than a week (see below).

Reporter expression was stable and both parts of the fusion reporter hNIS-mGFP were functional (Fig. 2, Fig. S3) with hNIS-mGFP^+^ HLCs detectable *in vivo* post intrahepatic administration (Fig. 3). A week post-administration traceable HLCs were undetectable, likely due to HLC death in our animal model, but traceable control tumour cells remained detectable for weeks when administered both subcutaneously and intra-hepatically demonstrating long-term tracking capability and that death of our HLC population is not due to the method of transplantation but rather survival of tumour cells is due to the inherent survival advantage of SK-Hep liver cancer cells (Fig. S4-S5). The chosen dual-mode reporter permits streamlined preclinical research (convenient cytometry and histology due to fluorescent protein presence) with the CGT10 line used in this study suitable for clinical translation, highlighting the potential of this system for pre-clinical *in vivo* tracking of cells with therapeutic potential. hNIS, without the mGFP fusion, is also suitable for clinical research (for SPECT and PET imaging) and is already used in clinical trials as a reporter gene in different contexts (DeGrado, 2016). Similar results were obtained in primary foetal hepatocytes (Fig. S2) indicating our findings are not limited to iPSC-derived HLCs.

To our knowledge this is the first study to introduce hNIS for non-invasive imaging of hiPSC-derived HLCs *in vivo*, and the first to do so during differentiation. Our results highlight the retention of the hepatic phenotype in LV-transduced immature HLCs throughout their maturation, with stable hNIS-mGFP reporter expression enabling their *in vivo* imaging. This strategy addresses the unmet need for a non-invasive *in vivo* tracking strategy for pre-clinical assessment of HLC transplantation including engraftment optimisation studies and provides a promising tool for future clinical research.

## Declaration of Competing Interest

Authors declare that they have no competing financial interests.

## CRediT authorship contribution statement

**Candice Ashmore-Harris:** Conceptualization, Data curation, Writing - original draft. **Samuel JI Blackford:** Conceptualization, Data curation. **Benjamin Grimsdell:** Data curation. **Ewelina Kurtys:** Data curation. **Marlies C Glatz:** Data curation. **Tamir S Rashid:** . **Gilbert O Fruhwirth:** Conceptualization, Data curation, Writing - original draft.

## Appendix Supplementary materials

Image, application 1

Image, application 2

Image, application 3

Image, application 4

Image, application 5

Image, application 6

Image, application 7
