# Supplementary material for: Reporter gene-engineering of human induced pluripotent stem cells during differentiation renders in vivo traceable hepatocyte-like cells accessible
Source: Stem Cell Res. 2019 Dec;41:101599. doi: 10.1016/j.scr.2019.101599 (PMC6905152; doi:10.1016/j.scr.2019.101599)
Supplement: Supplementary file 1 [file mmc1.pdf]

Supplementary Material

to the manuscript entitled *"Reporter gene-engineering of human induced pluripotent stem cells during differentiation renders in vivo traceable hepatocyte-like cells accessible."* by

Candice Ashmore-Harris, Samuel JI Blackford, Benjamin Grimsdell, Ewelina Kurtys, Marlies C Glatz, Tamir S Rashid, and Gilbert O Fruhwirth.

1) Supplementary Results

Impact of hNIS-mGFP reporter expression on fetal hepatocytes.

To verify that our results obtained in iPSC-derived HLCs were representative of primary cells, foetal hepatocytes, which are the closest primary cell type to iPSC-derived hepatocytes (Baxter et al., 2015), were transduced and similarly characterized as the HLCs above. Foetal hepatocytes did express hNIS-mGFP (Fig.S2A) with the reporter also being functional (Fig.S2B) They also secreted albumin at comparable levels to untransduced cells as determined by ELISA, although the absolute secretion levels varied depending on the cell quality and age of the fetus (Fig.S2C). Expression of a panel of mature and immature hepatocyte genes was compared between the transduced and control populations by quantitative real-time PCR (Fig.S2D). Primary hepatocytes are known to undergo dedifferentiation following a period of culture *ex vivo* (Rowe et al., 2013). This was reflected in the decreased fetal hepatocyte gene expression profile relative to mRNA expression of cells at the point of isolation. Importantly, observed changes were similar between reporter-expressing and control cells and unrelated to cell viability, which was also consistent between reporter-expressing and control cells (Fig.S2E). These results support our findings in HLCs suggesting that hNIS-mGFP reporter expression in hepatocyte populations does not adversely impact their hepatocyte phenotype.

2) Supplementary Figures

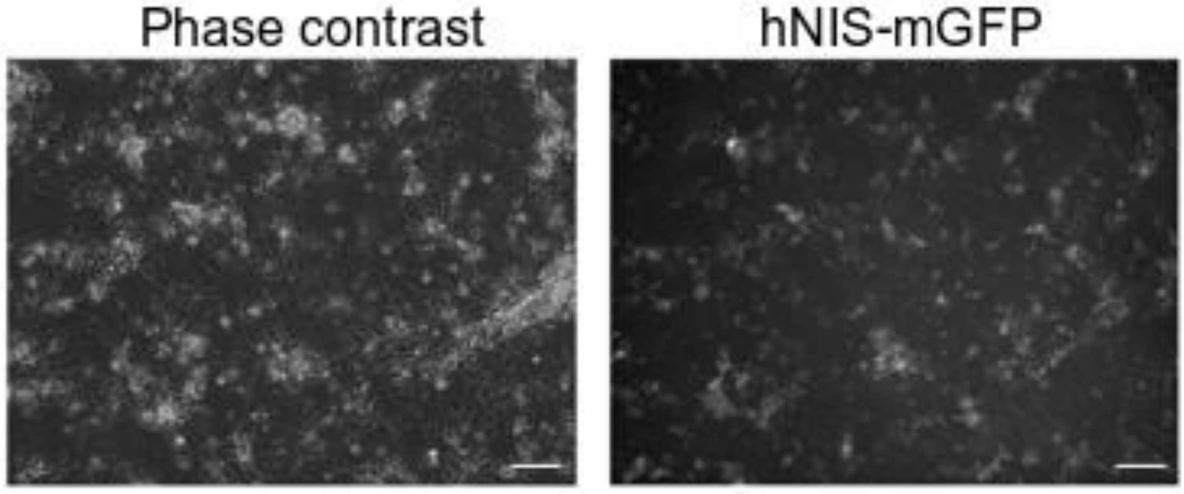

Supplementary Figure S1. Transduction of differentiating iPSCs at the hepatoblast stage is inefficient | iPSCs cells were differentiated as described (Sam paper). Here, on day 10 of the differentiation timeline, i.e. at the hepatoblast stage, cells were transduced with lentiviral particles (see Methods). Two days post transduction, cells were imaged live using an upright microscope equipped to obtain **(Left)** phase contrast and **(Right)** GFP fluorescence images. Phase contrast images show all differentiating cells while the GFP fluorescence images report on which cells were successfully transduced with lentiviral particles and then expressed the radionuclide-fluorescence reporter hNIS-mGFP. Transduction is inefficient and accompanied with significant cell loss as a consequence of the hepatoblast stage. We found transduction to be more efficient when performed on day 18 (see main manuscript). Scale bars are 100 µm.

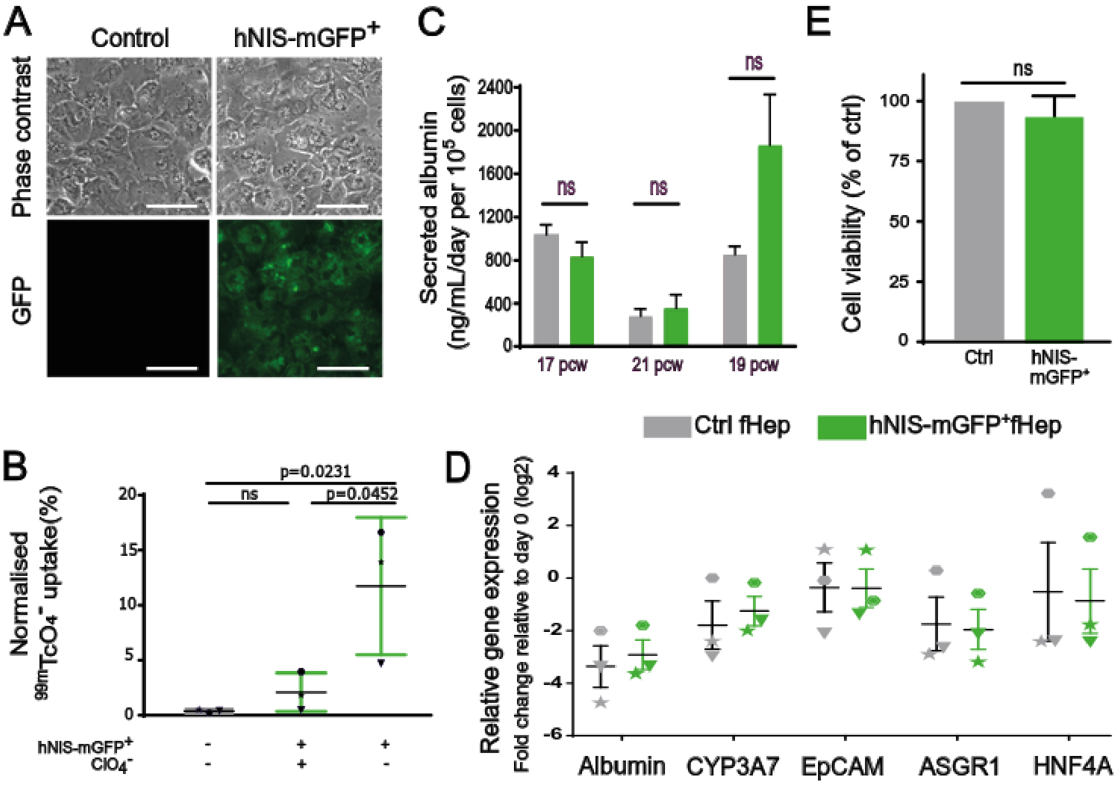

Supplementary Figure S2. hNIS-mGFP transduction does not adversely affect the hepatic phenotype of foetal hepatocytes. | (A) Morphology of control and transduced primary fetal hepatocytes. hNIS-mGFP$^+$ primary fetal hepatocytes (fHep) can be identified by correctly localized green fluorescence. Scale bar 100µm. **(B)** hNIS-mGFP function in transduced fetal hepatocytes (right) quantified by $^{99m}TcO_4^-$ uptake was compared to untransduced control fetal hepatocytes (left). Perchlorate inhibition was used to demonstrate uptake specificity and the expected reduction of radiotracer uptake in the presence of perchlorate was observed (middle). N=3 biological replicates (with three technical repeats per biological sample); error bars are SD. Differences between sample means a were analyzed by one-way ANOVA with Tukey's multiple comparison correctio; "ns" represents $p>0.05$. **(C)** Albumin production of fetal hepatocytes four days post-transduction measured by ELISA. Three different batches of fetal hepatocytes were obtained. Fetal hepatocytes were harvested at the indicated weeks post

conception (pcw). The mean albumin concentrations of each batch were compared by a two-tailed student's *t*-test; "ns" $p>0.05$; 3 technical repeats per population. Error bars are SEM. **(D)** qPCR analysis of hepatic mRNA expression four days post-transduction expressed as fold-change relative to expression at isolation (day 0). N=3 biological replicates (with four technical sample replicates each); error bars are SEM. Differences between mean fold-change for each gene were analyzed by two-tailed *t*-test. No significant differences were found ($p>0.05$) between control and hNIS-mGFP$^+$ fetal hepatocytes. Abbreviations: CYP3A7 is Cytochrome P450 Family 3A7; HNF4A is Hepatic nuclear factor 4α; ASGR1 is asialoglycoprotein receptor 1; EpCAM is Epithelial cell adhesion molecule. Symbols indicate the batches taken harvested at indicated weeks post conception (pcw): ⬣ = 17 pcw, ▼ = 21 pcw, ★ = 19 pcw. **(E)** Presto blue analysis of cell viability four days post transduction. Results normalized as a percent of control cell viability per sample. N=3 biological replicates (with three technical repeats per sample); error bars are SD. Data were compared using Students' *t*-test; "ns" $p>0.05$.

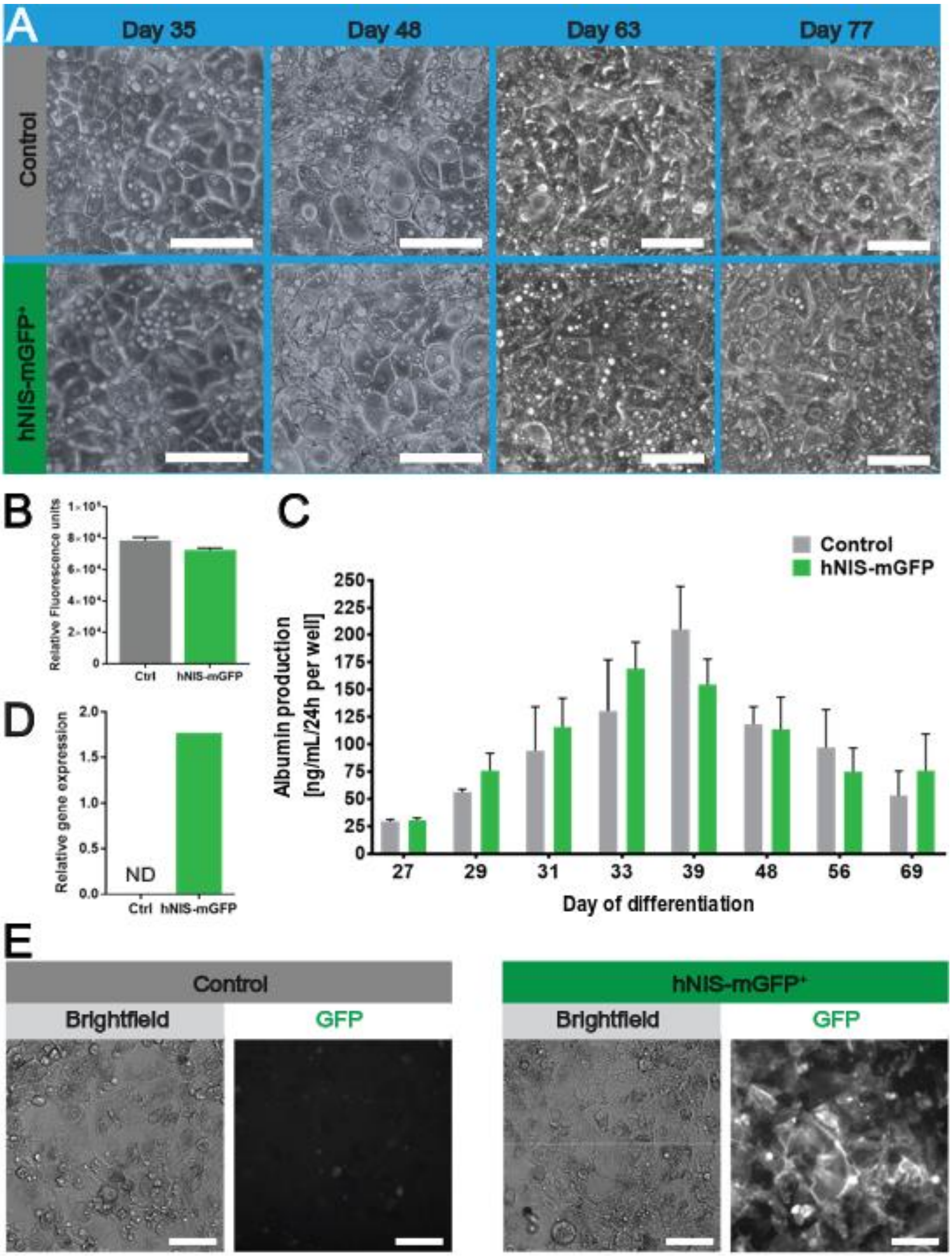

Supplementary Figure S3. Long-term culture of hNIS-mGFP^{+} HLCs *in vitro* demonstrated retention of reporter expression and of the hepatic phenotype as compared to untransduced HLCs. | (A) Representative micrographs showing the morphology of control and hNIS-mGFP^{+} A1AT HLCs over time (scale bars are 100 µm) **(B)** Presto blue analysis of cell viability at day 65 (N=3 replicate wells, error bars are SEM) demonstrating comparable

survival of untransduced and hNIS-mGFP^{+} HLCs **(C)** Albumin production of untransduced and hNIS-mGFP^{+} HLCs as a function of time in culture. Results obtained via ELISA indicated comparable retention (and eventual decline) of hepatic phenotype over a prolonged period of *in vitro* culture; N=3 replicate wells per population, error bars are SEM. **(D)** hNIS expression of HLCs collected at day 100 in culture as determined by qRT-PCR. Results are shown relative to expression at day 34 and demonstrate reporter expression is retained long-term. **(E)** Live cell fluorescence micrographs and corresponding brightfield images taken on a PerkinElmer Operetta at day 82 of culture. Micrographs demonstrate membranous GFP fluorescence as expected for hNIS-GFP (see also Fig.2A), corroborating results in (D); scale bars are 100 µm.

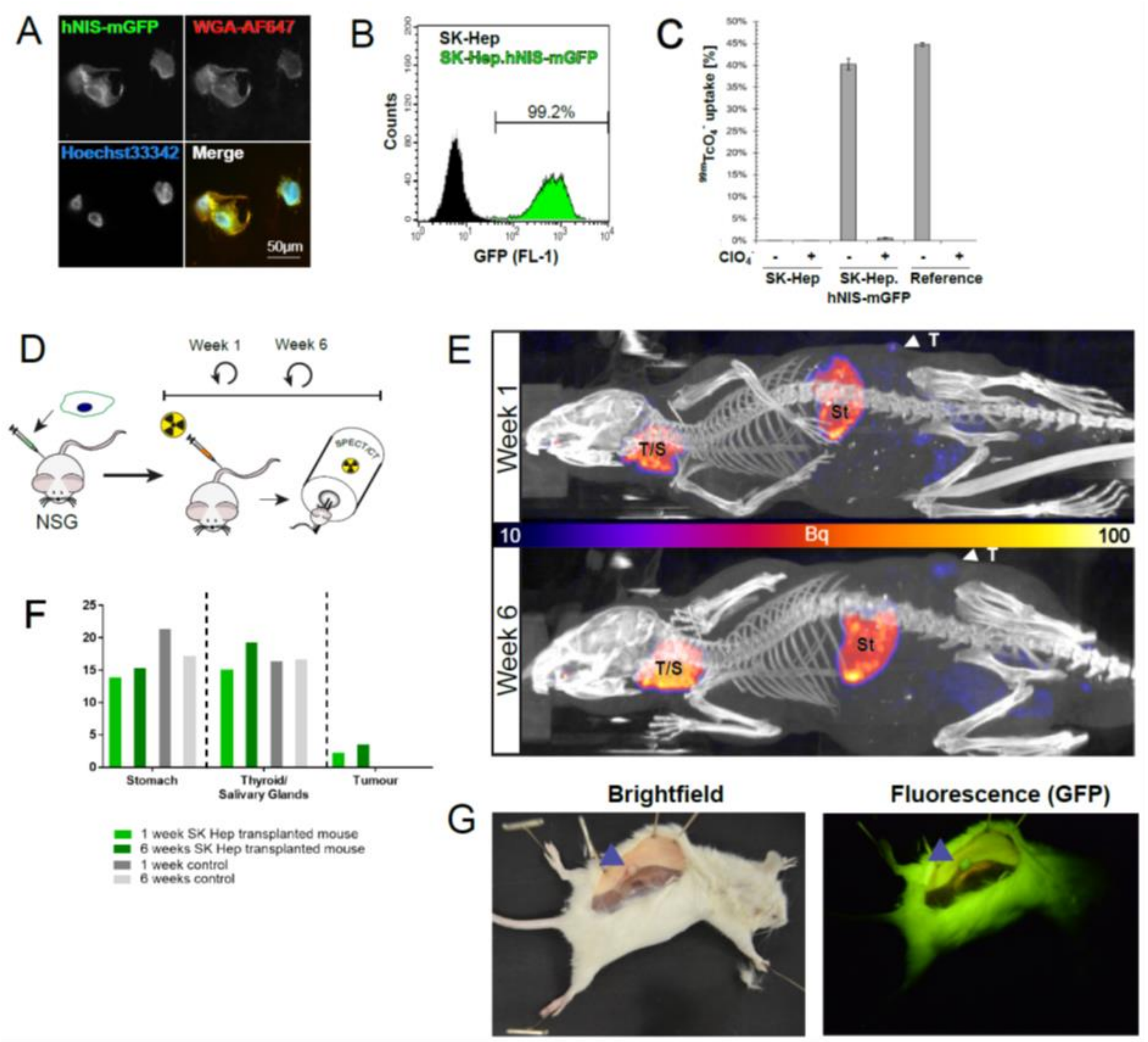

Supplementary Figure S4. Establishment and demonstration of *in vivo* tracking potential of human SK-Hep.hNIS-mGFP liver cancer cells. | SK-Hep cells were transduced with lentiviruses transferring hNIS-mGFP as previously described for other cancer cells (Volpe et al, 2018). Resulting cancer cells were FACS-sorted for purity and grown until stability. **(A)** Fluorescence microscopy showed co-localization of hNIS-GFP with the plasma membrane marker dye wheat germ agglutinin-Alexa647 (WGA-AF647). Representative cells are shown. Scale bar is 50 µm. **(B)** Flow cytometric analysis of FACS-sorted SK-Hep.hNIS-mGFP cells (green) as compared to parental SK-Hep cells (black) demonstrated >99% of cells were hNIS-mGFP^{+}. **(C)** hNIS-mGFP reporter gene function was quantified by $^{99m}TcO_4^-$ uptake assays. The NIS co-substrate perchlorate was used as a control for uptake specificity. **(D)** Scheme of the *in*

vivo experiment including subcutaneous tumour establishment and subsequent $^{99m}TcO_4^-$-afforded NIS imaging sessions by SPECT/CT. **(E)** NSG mice received SK-Hep.hNIS-mGFP tumour cells subcutaneously and were imaged one week following administration, with the administered cells at the injection site clearly detectable (white arrow; T). Expected endogenous signals were also detected (St: stomach; T/S: thyroid and salivary gland). After six weeks a small tumour was established, and its tumour cells continued to be *in vivo* traceable by SPECT/CT (white arrow; T). **(F)** Standard uptake values (SUV) from relevant tissues determined from *in vivo* images using VivoQuant (Invicro) software. **(G)** Post-mortem visualization of tumour fluorescence (arrow) using a torch excitation (450/20 nm BP) paired with an emission filter (500 nm LP) camera add-on (NightSea).

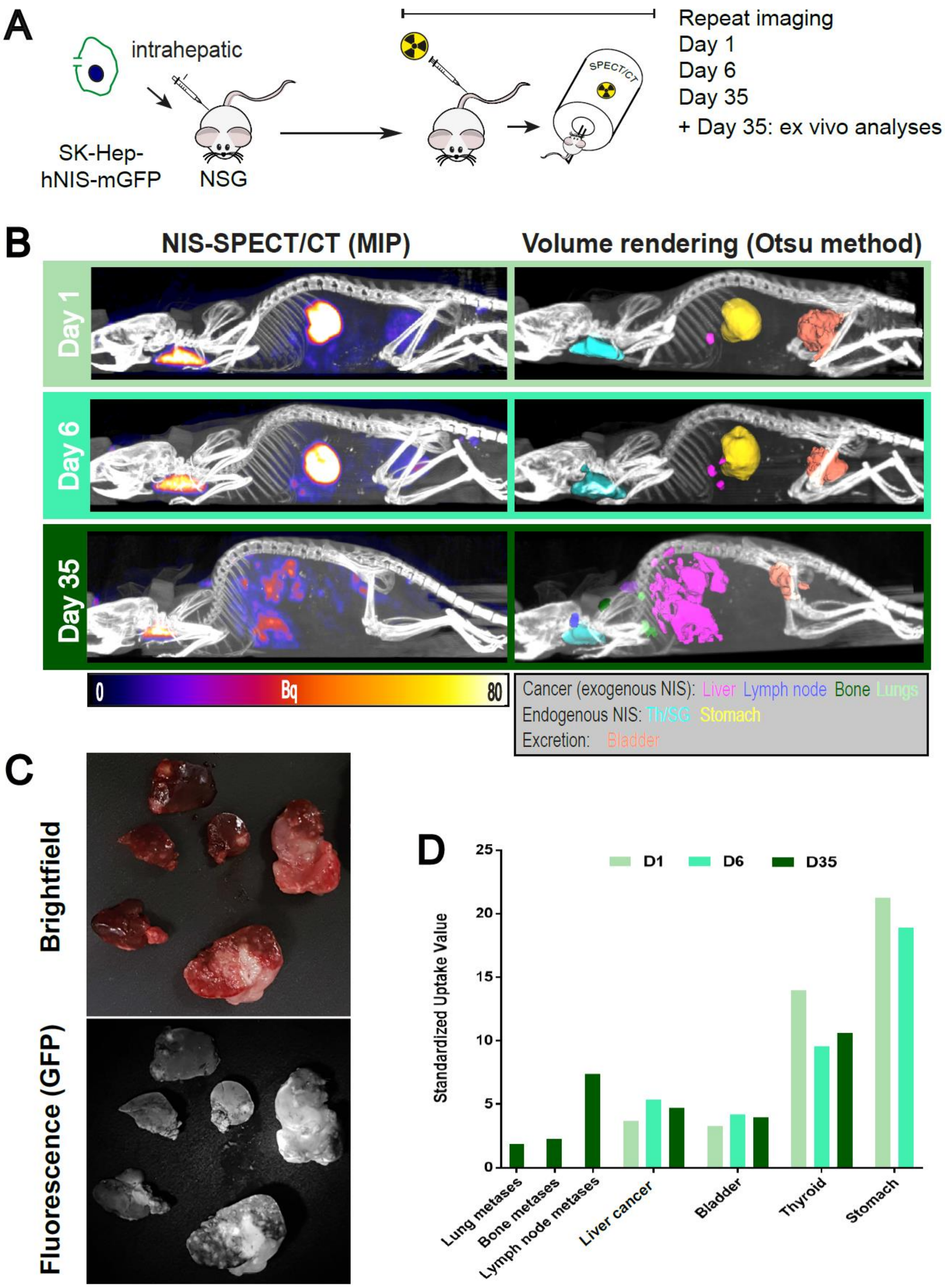

Supplementary Figure S5. Demonstration of longitudinal intra-hepatic *in vivo* tracking potential of human SK-Hep.hNIS-mGFP liver cancer cells. SK-Hep-hNIS-mGFP cells, as characterised in Fig.S4, were transplanted intra-hepatically to demonstrate that hNIS-mGFP^{+}

cells with an appropriate survival advantage are longitudinally trackable in the liver niche with retention of reporter function. **(A)** Scheme of the *in vivo* experiment including liver tumour establishment and subsequent $^{99m}TcO_4^-$-afforded NIS imaging sessions by SPECT/CT. **(B)** SK-Hep.hNIS-mGFP tumour cells were transplanted intra-hepatically into NSG mice and were imaged at days 1, 6 and 35 post transplantion. Administered cells at the transplant site are clearly detectable along with expected endogenous signals (stomach; thyroid and salivary gland). 3D volume rendering of SPECT signals subsequent to local signal/noise thresholding (Otsu method) are also overlaid onto the CT images at each timepoint to demonstrate the progression in cell volume and/or metastasis over time (yellow = stomach, cyan = thyroid, magenta = liver tumour, green = lung metastases, dark blue = lymph node metastasis, dark green = bone metastasis). **(C)** Standard uptake values (SUV) from relevant tissues and metastases determined from *in vivo* images using VivoQuant (Invicro) software. **(D)** Post-mortem visualization of liver tumour fluorescence using a torch excitation (450/20 nm BP) paired with an emission filter (500 nm LP) camera add-on (NightSea).

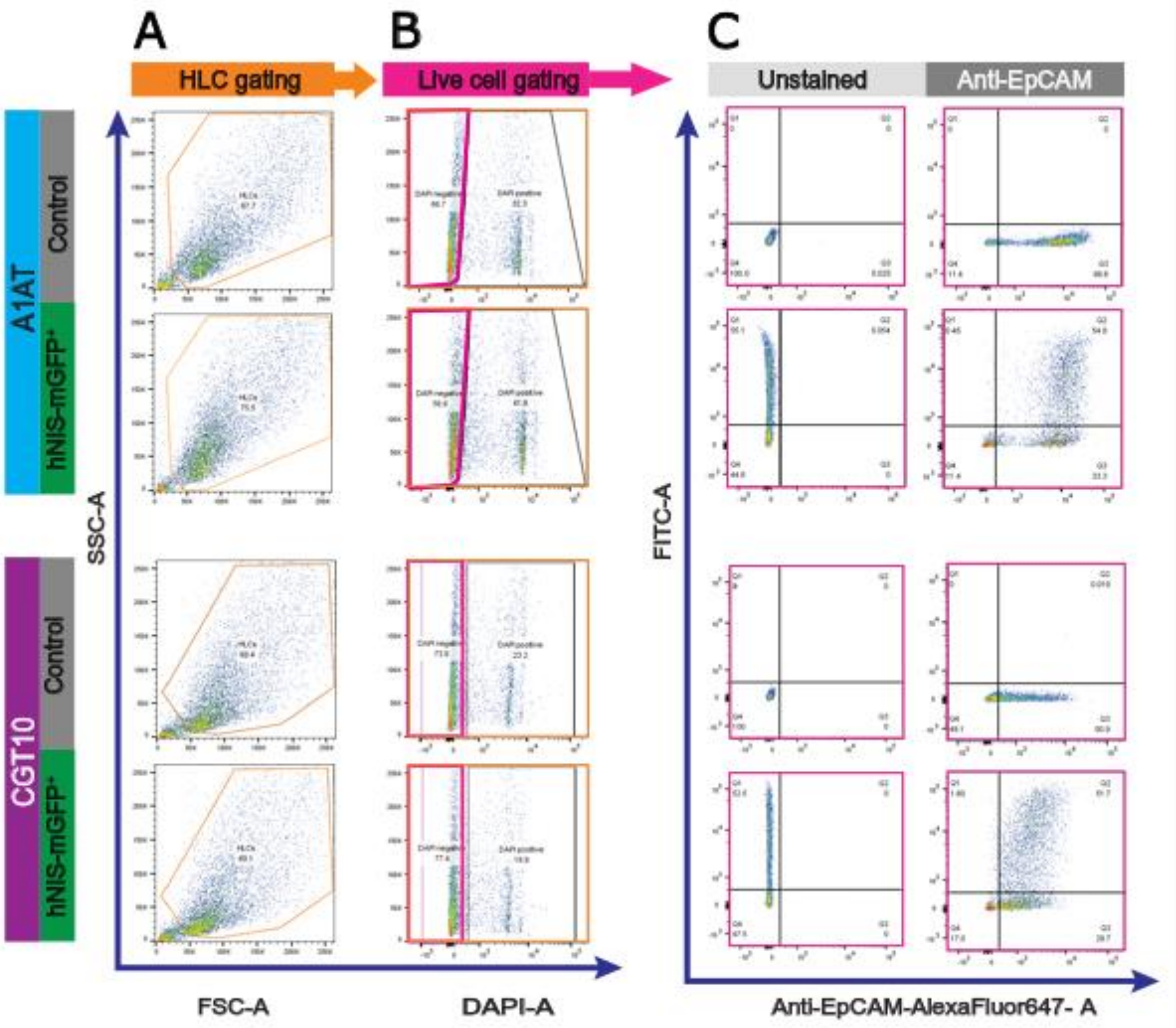

Supplementary Figure S6. Gating strategy for flow cytometric analyses of HLCs including typical data. | (A) Untransduced and hNIS-mGFP^{+} HLCs were first separated from debris on a FSC-A/SSC-A dot plot (accepted cells within yellow gate). **(B)** Live cells (negative for DAPI staining, pink gate) were then discriminated from dead cells (DAPI^{+}). **(C)** The gated live cell population was analyzed for hNIS-mGFP^{+} expression (by virtue of intrinsic GFP fluorescence, FITC channel) and stained with or without anti-EpCAM-AlexaFluor647 to identify the EpCAM^{+} population; analysis gates were set according to unstained controls (left).

3) Supplementary Methods

Cell isolation, culture and differentiation. HiPSC colonies from the patient-derived A1ATD$^{z/z}$ (Rashid et al., 2010; Yusa et al., 2011) or the cGMP derived CGT-RCiB-10 (WCB) hiPSC line (Cell and Gene Therapy Catapult, UK), herein referred to as A1AT and CGT10 respectively, were maintained in Essentia 8 Media on a layer of vitronectin (thin coating of 10 µg/mL solution in PBS applied for 1 hour at room temperature) at 37°C in a humidified atmosphere containing 5% (v/v) CO_2. hiPSCs were differentiated under hypoxic conditions (5% (v/v) O_2, 5% (v/v) CO_2, 37°C) via serial introduction of small molecules and growth factors as previously described (Blackford et al., 2018).

Human foetal liver tissues were obtained from the MRC/Wellcome-Trust funded Human Developmental Biology Resource (HDBR). The HDBR tissue bank is regulated and operated in line with the UK Human Tissue Authority (HTA) codes of practice and all required consent was obtained with approval (REC reference 08/H0712/34+5). Primary foetal hepatocytes were isolated from HDBR liver tissue obtained on the same day as termination following the principles outlined by Strom et al (Strom et al., 1996). Briefly, the liver was minced using a cell scraper or scalpel and the gallbladder and biliary tree removed. The minced liver was mixed thoroughly with Hank's Balanced Salt Solution (HBSS, Sigma) containing 0.1 M EGTA and centrifuged at 100g for 5 min. Supernatant was discarded, and the liver pellet washed in HBSS and centrifuged at 100g for 5 minutes. The pellet was resuspended in 25 mL Eagle's Minimum Essential Media (EMEM, Lonza) supplemented with 0.5 mg/mL collagenase XI (Sigma), 0.2 mg/ml DNase I (NEB) and penicillin/streptomycin. The suspension was heated at 37°C for 25 minutes, with periodic shaking and subsequently centrifuged (100g, 5 mins), washed in EMEM and centrifuged again (100g, 5mins). The resultant pellet was resuspended in fetal hepatocyte culture media [high glucose DMEM (4.5 mg/mL) supplemented with human insulin solution (50 µg/mL), Dexamethasone (40 nM/mL), 100IU penicillin/streptomycin, 2 mM *L*-glutamine

and 2% (v/v) non-essential amino acids], filtered through a 100 µm cell strainer and plated onto Collagen-I as with HLCs Cells were maintained at 37°C in a humidified atmosphere containing 5% (v/v) CO_2.

The rat mammary adenocarcinoma cell line MTLn3E.hNIS-TagRFP.Δ34-CXCR4.GFP (3E.Δ-NIS) served as a reference for reporter gene functional assays (Fruhwirth et al., 2014), and was cultured in αMEM supplemented with 5% (v/v) fetal bovine serum (FBS), 100IU/mL penicillin/streptomycin, 2 mM *L*-glutamine and 1 µg/mL puromycin. HepG2 cells (ATCC) were cultured in RPMI-1640 supplemented with 10% (v/v) FBS, 100IU/mL penicillin/streptomycin and 2 mM *L*-glutamine. Human embryonic kidney-derived 293T cells (ATCC) were grown in DMEM supplemented with 5% (v/v) foetal bovine serum (FBS), 100IU/mL penicillin/streptomycin, 2 mM *L*-glutamine and 1 µg/mL puromycin. All cell lines were maintained at 37°C in a humidified atmosphere containing 5% (v/v) CO_2.

Lentivirus production and titre analysis. Lentiviral particles were produced as previously described (Volpe et al., 2018). Briefly, 293T cells were co-transfected with the reporter gene plasmid pLNT/SFFV hNIS-mGFP and packaging plasmids and virus particles were harvested from cell supernatants 48 h after transfection and filtered through 0.45 µm filters to remove producer cells). Lentiviral particles were concentrated using standard centrifugation techniques (16,000g, 20 h) before being resuspended in phosphate buffered saline (PBS) and frozen at -80°C. Aliquots were thawed for transductions. Virus titre was determined using standard methodology with HepG2 liver cancer cells as target cells (see below).

Analysis of virus titre. To analyze the viral titre 2x10^{5} HepG2 cells were seeded per well in a 24-well plate. Serially diluted virus was applied in a final volume of 200 µL per well 24h after cell seeding. 24h post-infection the media was topped up to 500 µl. 48-72h post infection cells

were collected, washed in FACS buffer and acquired live on a BD FACS Calibur to assess the proportion of cells expressing GFP. Viral titre was calculated by the following equation:

$$Titre\ (Transducing\ Units/ml\ ) = \{(F\ x\ Cn)/V\}\ x\ DF$$

where F = GFP^{+} cell frequency, Cn = seeded cell number, V = viral inoculum volume and DF = dilution factor. Viral dilutions yielding GFP expression in the linear range (1-20%) were averaged to calculate the titer.

Cell viability determination of fetal hepatocytes. Presto Blue was used according to the manufacturer's instructions. Fluorescence was measured on a GloMax Discover multi-well plate reader (Promega) with 520±20 nm BP excitation and 600±20 nm BP emission filters.

Albumin determination. Albumin secretion into culture media or blood was assessed by ELISA using the Human Albumin Quantitation Set (Bethyl Laboratories) according to the manufacturer's instructions.

Quantitative real-time PCR. Cellular RNA extraction was performed using the TRIzol reagent (Invitrogen) according to manufacturers' instructions. cDNA was synthesized by reverse transcription using the SuperScript VILO cDNA Synthesis Kit (Invitrogen) with 500 ng of sample RNA according to the manufacturer's instructions. mRNA expression was assessed by qRT-PCR using the TaqMan Gene Expression Master Mix (Thermo-Fisher; for fetal hepatocytes) or iTaq Universal SYBR Green Supermix (Bio-Rad; for HLCs) on a CFX384 Real-Time PCR System (Bio-Rad) following manufacturers' instructions. For qPCR primers/probes used, refer to Supplementary Table.1 below. Quadruplicate technical replicates were performed. Sample threshold cycle *(C_t)* values were normalized to the human housekeeping gene β-Actin (*ACTB)*. Quantification was performed using the $\Delta\Delta C_t$ method and

relative gene expression for each independent differentiation was normalized to the corresponding control, untransduced population HLCs.

Supplementary Table.1: List of PCR primers and Taqman probes used in this study.

Housekeeper (SYBR)	
**β-actin Fwd**	CTTCCTTCCTGGGCATGG
**β-actin Rvs**	GTACAGGTCTTTGCGGATGT
**Hepatic primers (SYBR)**	
**α-fetoprotein Fwd**	TGAATCCAGAACACTGCATAGAA
**α-fetoprotein Rvs**	TATGGTAGCCAGGTCAGCTA
**Albumin Fwd**	CGTCGAGATGCACACAAGA
**Albumin Rvs**	GATACTGAGCAAAGGCAATCAAC
**ASGR1 Fwd**	GGCCTGAGAGAGACGTTCAG
**ASGR1 Rvs**	TCTCCAGCTGGGACTCTAGC
**CYP3A7 Fwd**	GTCAGCCTGATACTCCTCTATCT
**CYP3A7 Rvs**	TCAAACGTCCAATAGCCCTTAC
**EpCAM Fwd**	GCTCTGAGCGAGTGAGAACC
**EpCAM Rvs**	AACGCGTTGTGATCTCCTTC
**HNF4α Fwd**	GTGGTGGACAAAGACAAGAG
**HNF4α Rvs**	TGGACGGCTTCCTTCTT

Taqman Probes (SYBR)	
**β-actin VIC**	Hs01060665
**Albumin FAM**	Hs00609411
**CYP3A7 FAM**	Hs00426361
**EpCAM FAM**	Hs00158980
**ASGR1 FAM**	Hs01005019
**HNF4 α FAM**	Hs00230853
**C/EBPα FAM**	Hs00269972

Flow cytometry. hNIS-mGFP and EpCAM expression analyses were conducted in immature HLCs to assess transduction success and confirm the presence of a hepatic progenitor population. PBS-washed HLCs were incubated in TrypLE Select (37°C, 20 min), quenched with Hepatozyme and suspended as single cells by straining through a 100 µm filter. Cells were washed once in FACS buffer (PBS, 3% (w/v) BSA, 0.1 mM EDTA), incubated for 30min on ice with 0.5 µg/mL mouse anti-human EpCAM (CD326) antibody conjugated to

AlexaFluor647 (Biolegend; clone 9C4) before two washes (FACS buffer, centrifugation at 300g for 3 min). DAPI was added to stained cell suspensions (3 µM) prior to acquisition on a BD FACS CANTO II flow cytometer. Single stained beads (BD CompBead) with mouse anti-human CD31 antibody conjugated to FITC (Biolegend; clone WM59) or mouse anti-human EpCAM antibody conjugated to AlexaFluor647 (Biolegend; clone 9C4) were used for compensation. The gating strategy is detailed in Fig.S6. Results were analyzed using FlowJo v10 (Treestar).

***In vitro* radio tracer uptake in hNIS-mGFP expressing cells.** hNIS function was assessed by the ability to take up the radiotracer $^{99m}TcO_4^-$. $^{99m}TcO_4^-$ was generator-produced and supplied in phosphate buffered saline (PBS) on the day of experimentation by the in-house radiopharmacy. Where sensitivity to competition with the hNIS co-substrate perchlorate was required cells were incubated with PBS containing Ca^{2+} and Mg^{2+} (PBS^{++}) supplemented with 12.5 µM $NaClO_4$ for 20 min at 37°C, 5% CO_2 prior to the start of the assay. Cells were washed in PBS^{++} and incubated with 50 kBq/mL $^{99m}TcO_4^-$ at 37°C, 5% CO_2 for 30 min. Wells were washed twice with 700 µL PBS^{++} and cells lifted by trypsinization (TrypLE). Radioactivity in supernatants, washes and resulting cell pellets was determined using a γ-counter (1282 Compugamma, LKB-Wallac). The percentage of the administered activity taken up by the cells was calculated (Equ.1). Blank wells, and cell-free collagen-I coated wells were used as background controls (confirmed negligible in all experiments). 3E-Δ-NIS cells were used as a positive assay control ($3x10^5$ cells/well in a 12-well plate) and for inter-assay normalization.

$$\%Activity = \frac{Counts\ per\ minute[Cells]}{Counts\ per\ minute[Cells]+Counts\ per\ minute[Supernatant/Wash]} \qquad \text{(Equ.1)}$$

Immunofluorescence staining. Transduced or control HLC populations passaged at day 21 of differentiation were seeded at a density of $7x10^4$ cells per well on a black µClear 96-well plate

(Cellstar) coated with collagen-I and matured under standard conditions until day 34. Fixing (4% (w/v) paraformaldehyde in PBS, 15 min), blocking and staining were performed at room temperature. For nuclear staining (HNF4A) cells were permeabilized using 0.5% (v/v) Triton X-100 in PBS for 10 min before being washed twice with PBS and blocked (PBS containing 1% (w/v) BSA, 3% (w/v) donkey serum and 0.1% (w/v) Triton-100) for 20 min. Cells were stained in blocking solution containing goat anti-human albumin (10 µg/mL; Bethyl Laboratories; A80-129A) and rabbit anti-human HNF4α (1.48 µg/mL; Abcam; clone EPR3648) primary antibodies for 1 hour. Cells were washed with PBS and incubated for 40 min in the dark with donkey anti-goat AlexaFluor647 and donkey anti-rabbit AlexaFluor568 (both 3 µg/mL). Nuclei were stained with DAPI (NucBlue; 40 µL/mL) prior to imaging with a PerkinElmer Operetta automated high content microscope equipped with fluorescence filter settings appropriate for all fluorophores used.

4) Supplementary References

Baxter, M., Withey, S., Harrison, S., Segeritz, C.P., Zhang, F., Atkinson-Dell, R., Rowe, C., Gerrard, D.T., Sison-Young, R., Jenkins, R., et al. (2015). Phenotypic and functional analyses show stem cell-derived hepatocyte-like cells better mimic fetal rather than adult hepatocytes. J. Hepatol. *62*, 581–589.

Blackford, S.J., Ng, S.S., Segal, J.M., King, A., Moore, J., Sheldon, M., Ilic, D., Dhawan, A., Mitry, R., and Rashid, T.S. (2018). Validation of a library of cGMP-compliant human pluripotent stem cell lines for use in liver therapy. Stem Cells Transl. Med.

Fruhwirth, G.O., Diocou, S., Blower, P.J., Ng, T., and Mullen, G.E.D. (2014). A Whole-Body Dual-Modality Radionuclide Optical Strategy for Preclinical Imaging of Metastasis and Heterogeneous Treatment Response in Different Microenvironments. J. Nucl. Med. *55*, 686–694.

Rashid, S.T., Corbineau, S., Hannan, N., Marciniak, S.J., Miranda, E., Alexander, G., Huang-doran, I., Griffin, J., Ahrlund-richter, L., Skepper, J., et al. (2010). Modeling inherited metabolic disorders of the liver using human induced pluripotent stem cells. J. Clin. Invest. *120*, 3127–3136.

Rowe, C., Gerrard, D.T., Jenkins, R., Berry, A., Durkin, K., Sundstrom, L., Goldring, C.E., Park, B.K., Kitteringham, N.R., Hanley, K.P., et al. (2013). Proteome-wide analyses of human hepatocytes during differentiation and dedifferentiation. Hepatology *58*, 799–809.

Strom, S.C., Pisarov, L.A., Dorko, K., Thompson, M.T., Schuetz, J.D., and Schuetz, E.G. (1996). Use of human hepatocytes to study P450 gene induction. Methods Enzymol. *272*, 388–401.

Volpe, A., Man, F., Lim, L., Khoshnevisan, A., Blower, J., Blower, P.J., and Fruhwirth, G.O. (2018). Radionuclide-fluorescence Reporter Gene Imaging to Track Tumor Progression in Rodent Tumor Models. J. Vis. Exp. e57088–e57088.

Yusa, K., Rashid, S.T., Strick-Marchand, H., Varela, I., Liu, P.-Q.Q., Paschon, D.E., Miranda, E., Ordóñez, A., Hannan, N.R.F., Rouhani, F.J., et al. (2011). Targeted gene correction of α1-antitrypsin deficiency in induced pluripotent stem cells. Nature *478*, 391–394.
