# Supplementary figures and images for: Reporter gene-engineering of human induced pluripotent stem cells during differentiation renders in vivo traceable hepatocyte-like cells accessible
Source: Stem Cell Res. 2019 Dec;41:101599. doi: 10.1016/j.scr.2019.101599 (PMC6905152; doi:10.1016/j.scr.2019.101599)

Phase contrast

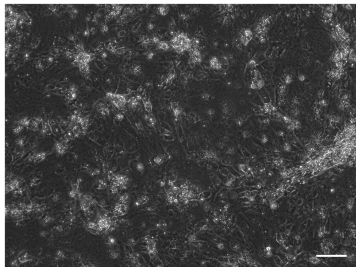

hNIS-mGFP

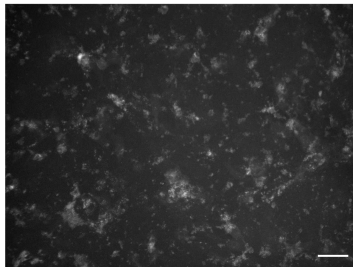

Supplement: Supplementary file 2 [file mmc2.pdf]

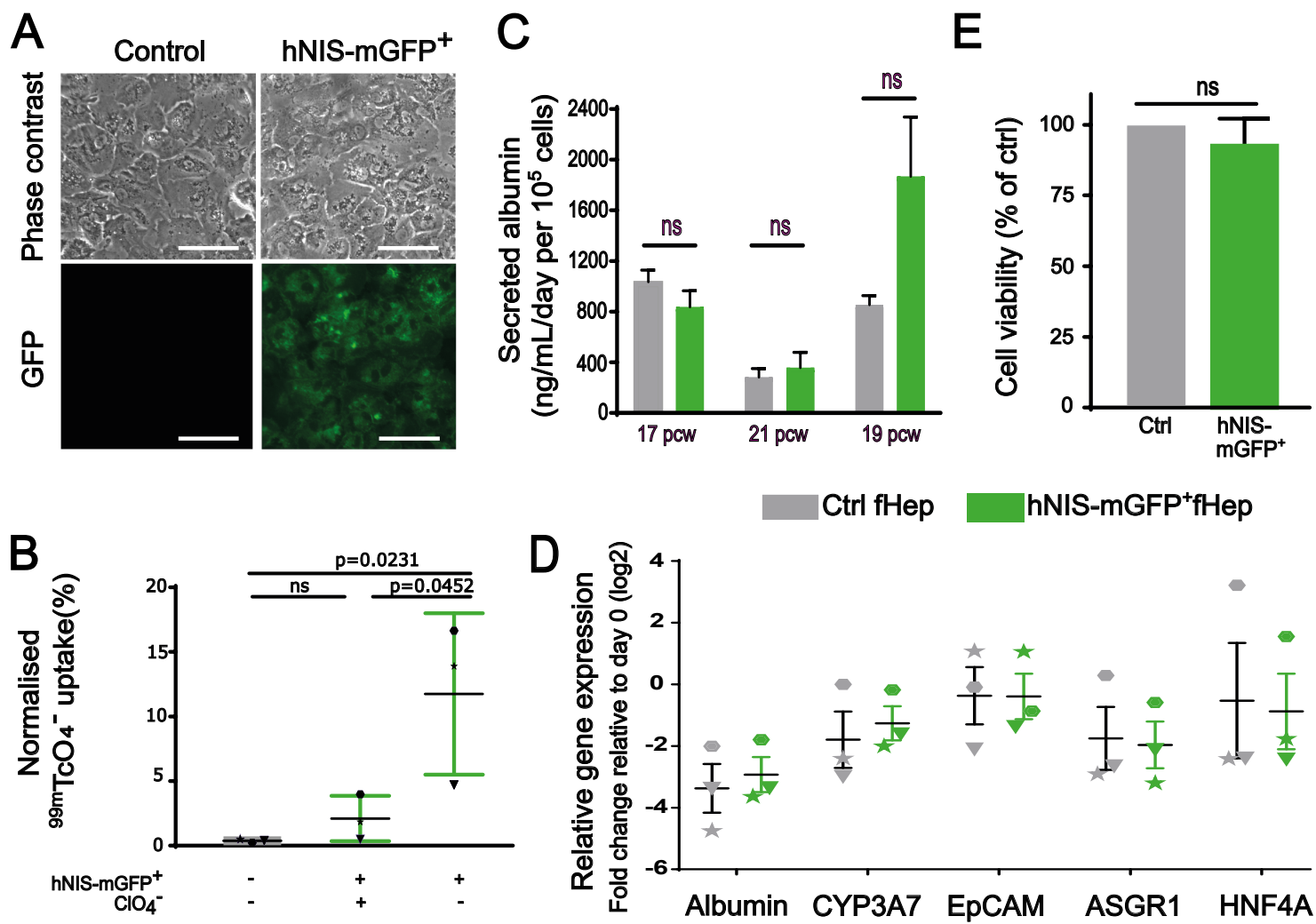

Supplement: Supplementary file 3 [file mmc3.pdf]

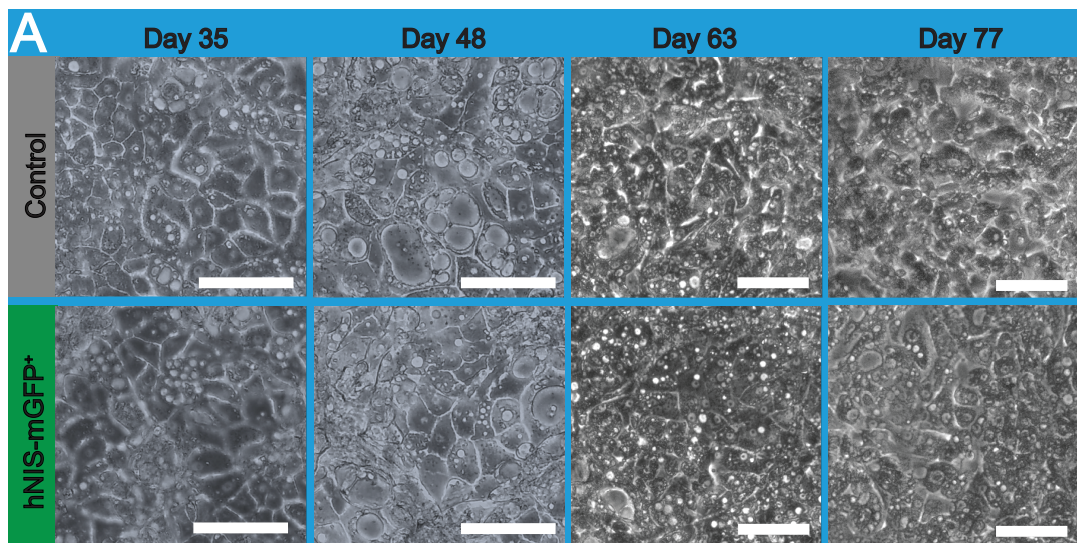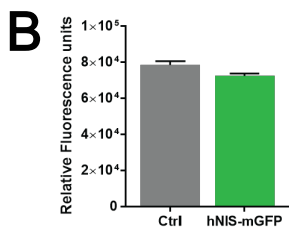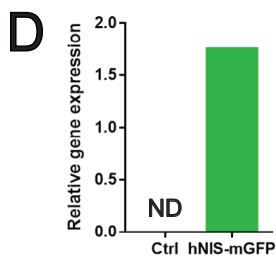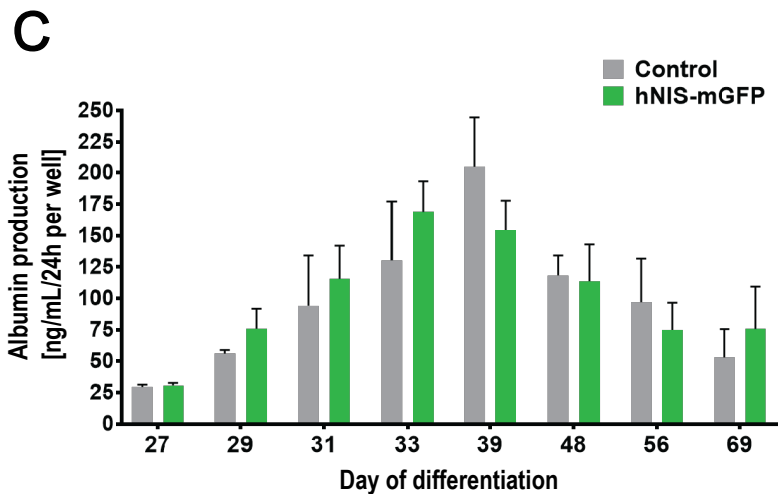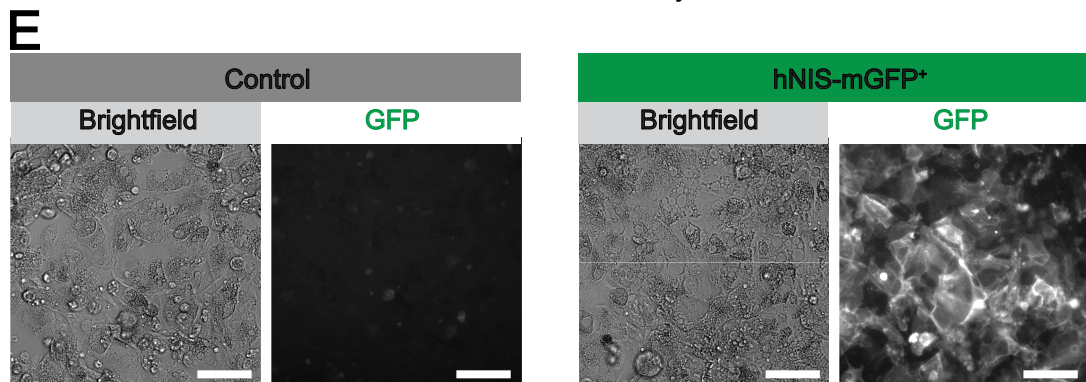

Supplement: Supplementary file 4 [file mmc4.pdf]

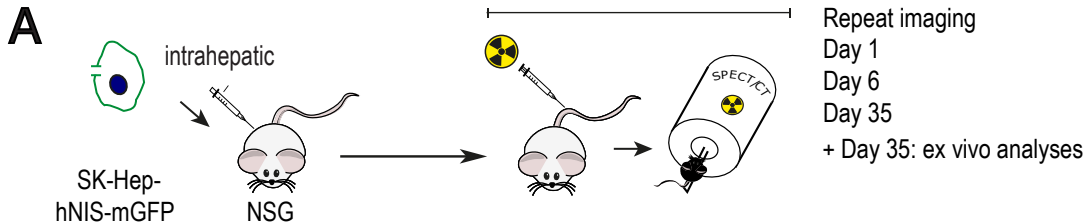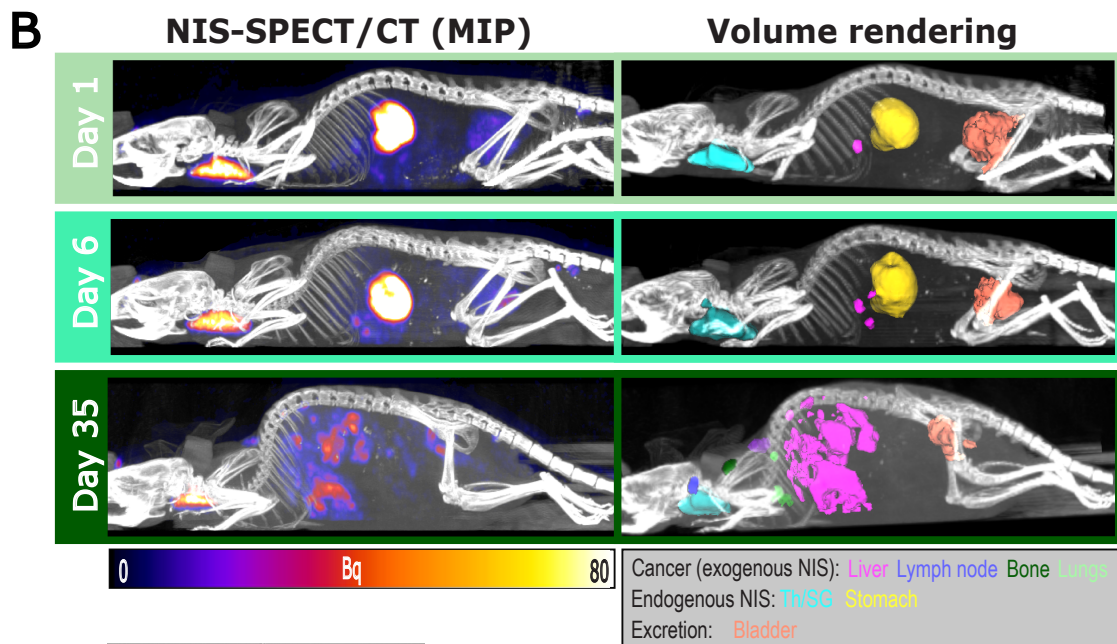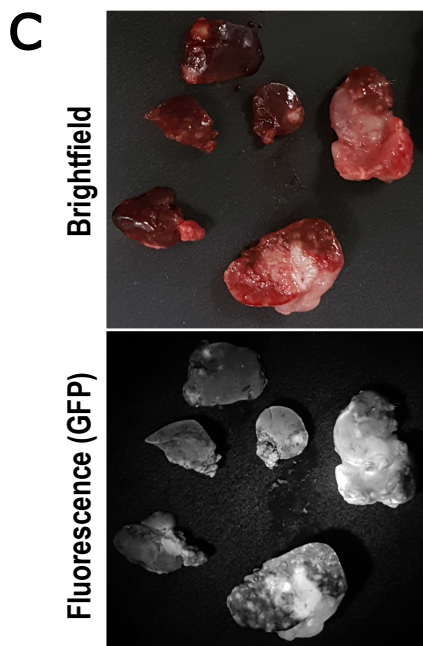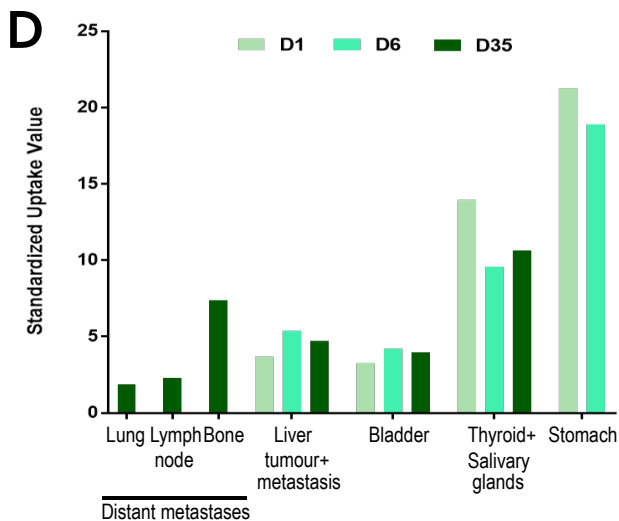

Supplement: Supplementary file 6 [file mmc6.pdf]
