# Supplementary material for: Reporter gene-engineering of human induced pluripotent stem cells during differentiation renders in vivo traceable hepatocyte-like cells accessible
Source: Stem Cell Res. 2019 Dec;41:101599. doi: 10.1016/j.scr.2019.101599 (PMC6905152; doi:10.1016/j.scr.2019.101599)
Supplement: Supplementary file 5 [file mmc5.pdf]

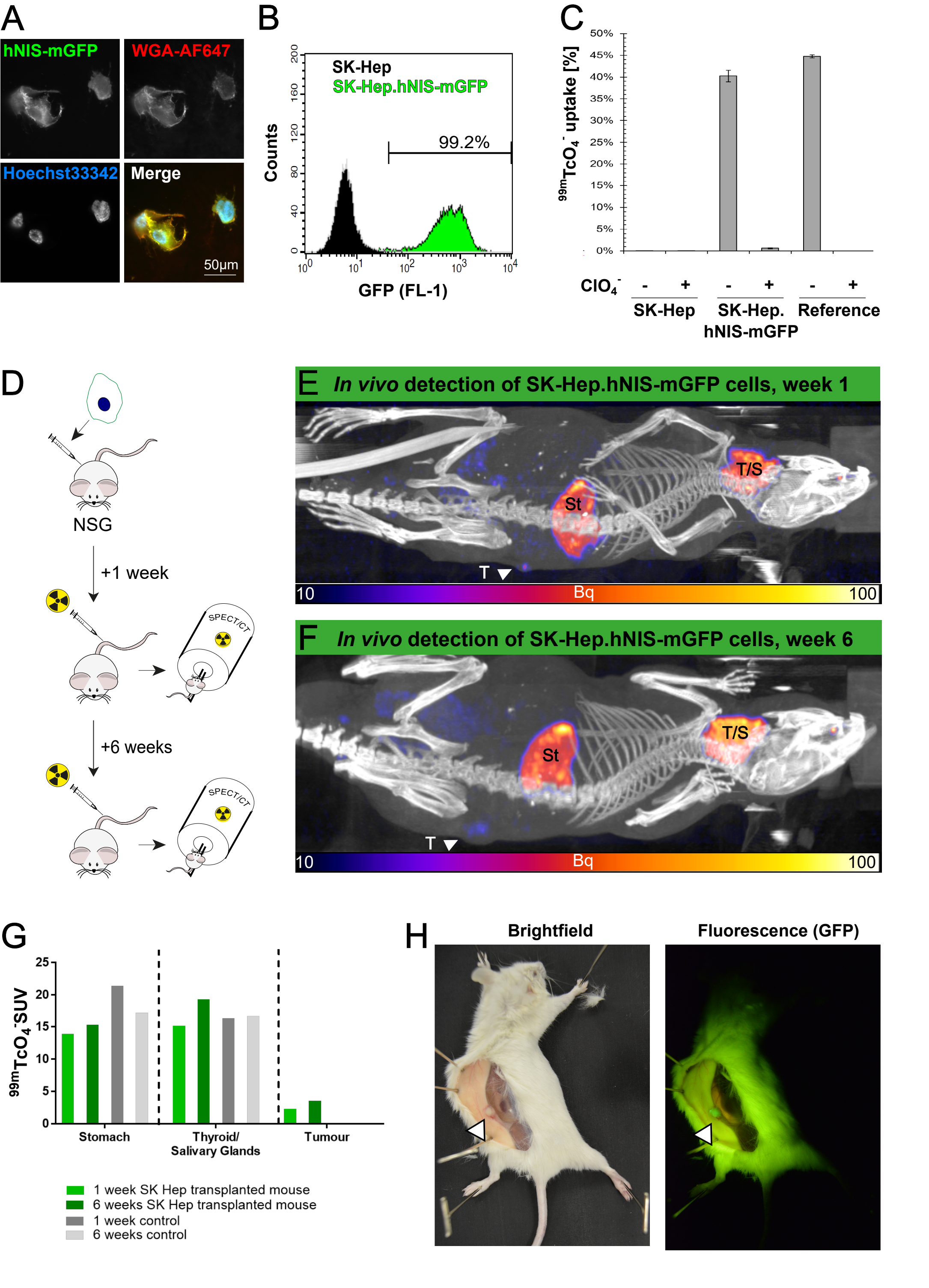

A
hNIS-mGFP
WGA-AF647
Hoechst33342
Merge
50µm
B
SK-Hep
SK-Hep.hNIS-mGFP
99.2%
Counts
GFP (FL-1)
C
99mTcO4- uptake [%]
ClO4-
SK-Hep
SK-Hep. hNIS-mGFP
Reference
D
NSG
+1 week
SPECT/CT
+6 weeks
E In vivo detection of SK-Hep.hNIS-mGFP cells, week 1
T/S
St
T
10
Bq
100
F In vivo detection of SK-Hep.hNIS-mGFP cells, week 6
G
99mTcO4- SUV
Stomach
Thyroid/ Salivary Glands
Tumour
1 week SK Hep transplanted mouse
6 weeks SK Hep transplanted mouse
1 week control
6 weeks control
H
Brightfield
Fluorescence (GFP)
