# Supplementary material for: Reporter gene-engineering of human induced pluripotent stem cells during differentiation renders in vivo traceable hepatocyte-like cells accessible
Source: Stem Cell Res. 2019 Dec;41:101599. doi: 10.1016/j.scr.2019.101599 (PMC6905152; doi:10.1016/j.scr.2019.101599)
Supplement: Supplementary file 7 [file mmc7.pdf]

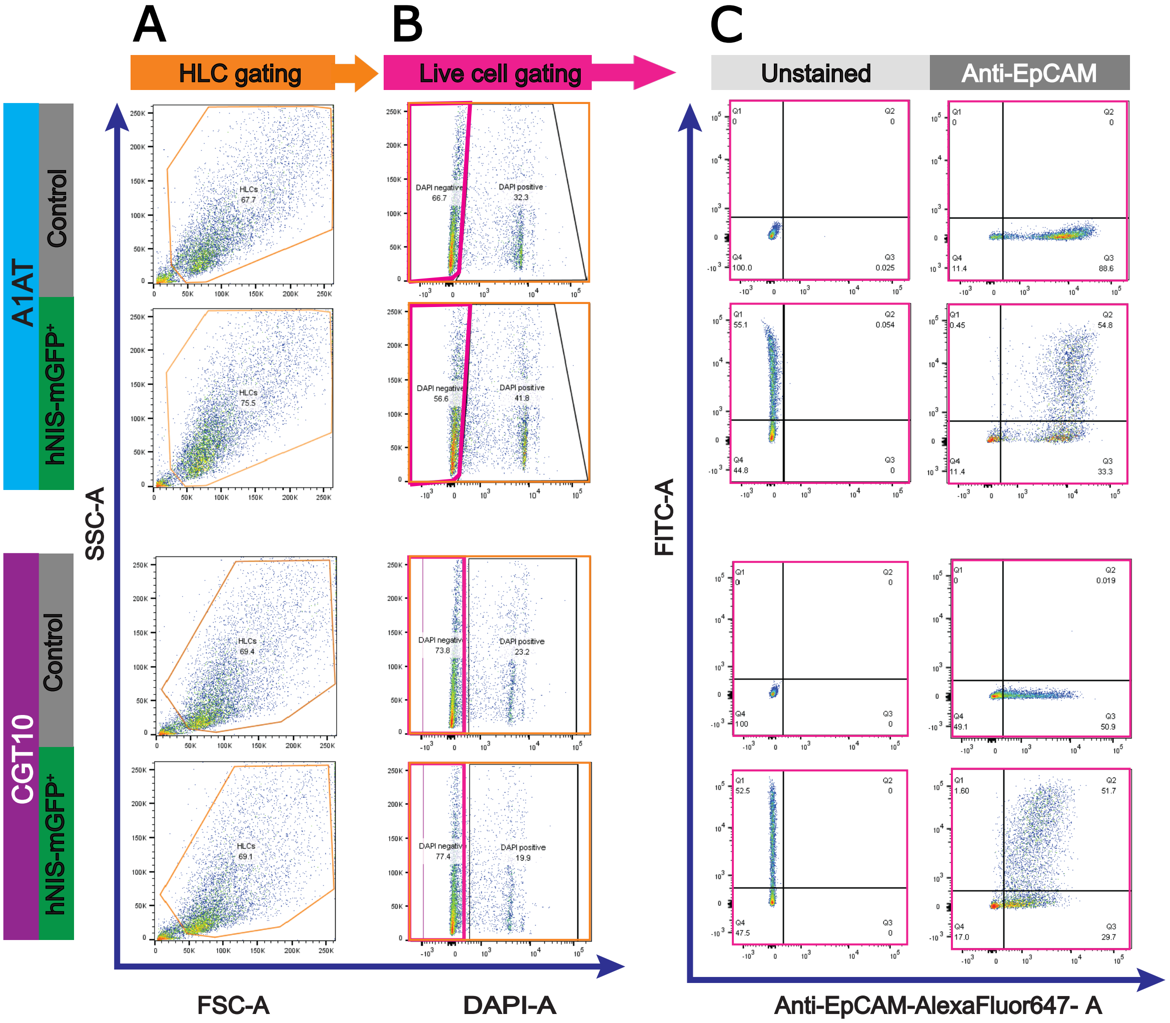
A
B
C
HLC gating
Live cell gating
Unstained
Anti-EpCAM
A1AT
Control
hNIS-mGFP+
CGT10
Control
hNIS-mGFP+
SSC-A
FITC-A
HLCs 67.7
HLCs 75.5
HLCs 69.4
HLCs 69.1
DAPI negative 66.7
DAPI positive 32.3
DAPI negative 56.6
DAPI positive 41.8
DAPI negativ 73.8
DAPI positive 23.2
DAPI negativ 77.4
DAPI positive 19.9
Q1 0
Q2 0
Q4 100.0
Q3 0.025
Q1 0
Q2 0
Q4 11.4
Q3 88.6
Q1 55.1
Q2 0.054
Q4 44.8
Q3 0
Q1 0.45
Q2 54.8
Q4 11.4
Q3 33.3
Q1 0
Q2 0
Q4 100
Q3 0
Q1 0
Q2 0.019
Q4 49.1
Q3 50.9
Q1 52.5
Q2 0
Q4 47.5
Q3 0
Q1 1.60
Q2 51.7
Q4 17.0
Q3 29.7
FSC-A
DAPI-A
Anti-EpCAM-AlexaFluor647- A
